# Generation of pyridyl coordinated organosilicon cation pool by oxidative Si-Si bond dissociation

**DOI:** 10.1186/1860-5397-3-7

**Published:** 2007-02-08

**Authors:** Toshiki Nokami, Ryoji Soma, Yoshimasa Yamamoto, Toshiyuki Kamei, Kenichiro Itami, Jun-ichi Yoshida

**Affiliations:** 1 Department of Synthetic Chemistry and Biological Chemistry, Graduate School of Engineering, Kyoto University, Kyotodaigakukatsura, Nishikyo-ku, Kyoto, 615-8510, Japan

## Abstract

An organosilicon cation stabilized by intramolecular pyridyl coordination was effectively generated and accumulated by oxidative Si-Si bond dissociation of the corresponding disilane using low temperature electrolysis, and was characterized by NMR and CSI-MS.

## Findings

**Scheme 1:**
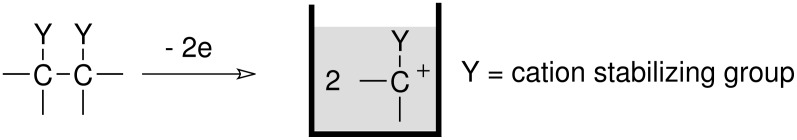
Electrochemical generation of carbocations by oxidative C-C bond dissociation.

We have recently developed the "cation pool" method, which involves the irreversible oxidative generation and accumulation of highly reactive cations in the absence of nucleophiles [1–5]. Heteroatom-stabilized carbocations, such as *N*-acyliminium ion pools and alkoxycarbenium ion pools have been generated based on oxidative C-H, C-Si, and C-S bond dissociation. Very recently, the oxidative C-C bond dissociation has been found to be effective for generation of a pool of a carbocation having a stabilizing group as shown in Scheme 1[6].

**Scheme 2:**
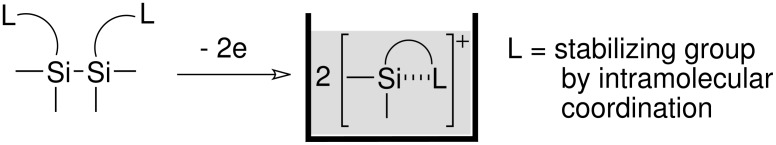
Electrochemical generation and accumulation of organosilicon cation by oxidative Si-Si bond dissociation.

We have been interested in generation and accumulation of cations of other elements such as silicon using the "cation pool" method. Organosilicon cations are known to be extremely unstable and difficult to accumulate in solution [7–11]. Organosilicon cations having appropriate donor ligands are, however, reasonably stable to accumulate in solution and many examples of such donor-stabilized organosilicon cations have been reported in the literature [12–18]. Herein, we report the generation and accumulation of a donor-stabilized organosilicon cation by the electrochemical oxidative Si-Si bond dissociation (Scheme 2) [19–21].

Symmetrical disilanes having coordinating groups on both silicon atoms were used as starting materials for electrochemical generation and accumulation of organosilicon cations, because oxidative dissociation of the Si-Si bond leads to the formation of two equivalents of organosilicon cations and no other product is formed.

In our earlier study, it was found that the introduction of a coordinating group such as a pyridyl group decreased the oxidation potential of tetraalkylstannanes, although there is no indication of the coordination in the neutral molecule. Dynamic intramolecular coordination to tin seems to facilitate electron transfer [22]. The coordination also stabilizes the thus-generated radical cation and weakens the C-Sn bond. A similar effect of intramolecular coordination was observed in the case of silicon [23]. Another important point is that pyridyl group is rather inactive toward the anodic oxidation. Thus, we chose to use a pyridyl group as a donor ligand.

**Figure 1:**
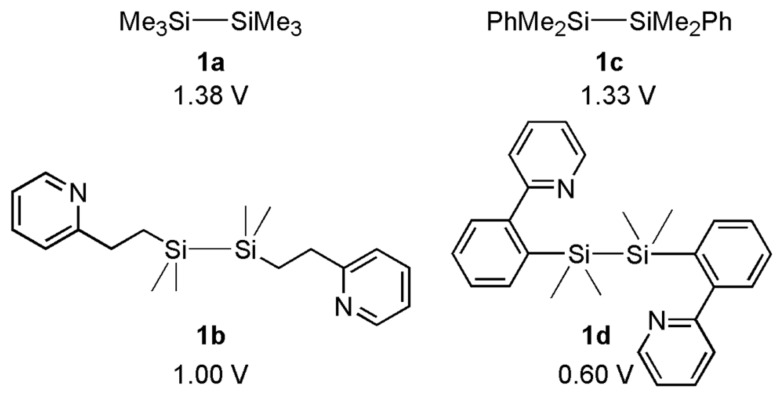
Oxidation potentials (E_d_; decomposition potential) of disilanes determined by rotating disk electrode voltammetry (RDE) in Bu_4_NClO_4_/CH_3_CN.

First, we prepared disilanes having pyridyl groups in appropriate positions and measured their oxidation potentials [24–25]. The oxidation potential of 2-pyridylethyl substituted disilane **1b** was slightly less positive than hexamethyldisilane **1a**. On the other hand, the oxidation potential of 2-pyridylphenyl substituted disilane **1d** was much less positive than the corresponding disilane **1c** having phenyl groups (Figure 1).

^29^Si NMR chemical shifts of **1b** and **1d** were similar to those of **1a** and **1c**, indicating that no coordination of the pyridyl groups on silicon existed in the neutral molecules (Supporting Information File 1). Therefore, the significant effect of the 2-pyridyl group on the oxidation potential may be ascribed to effective intramolecular coordination to stabilize the radical cation intermediate. The conformationally less flexible 2-pyridylphenyl group seems to be more effective than the 2-pyridylethyl group.

**Figure 2:**
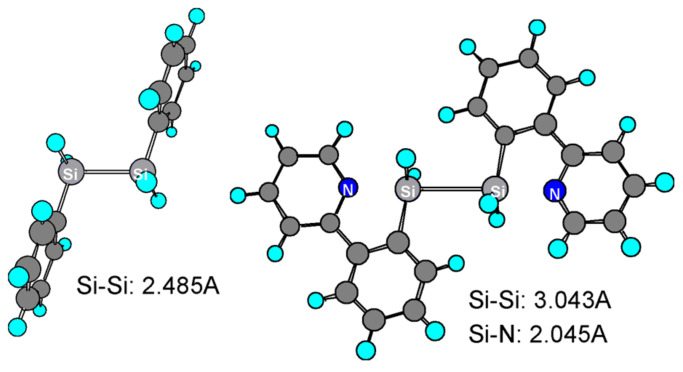
Optimized structures of radical cations of 1,2-diphenyldisilane and 1,2-bis [*o*-(2-pyridyl)- phenyl]disilane obtained by DFT calculation (B3LYP/LANL2DZ).

The intramolecular coordination in the radical cation is supported by the DFT calculations as shown in Figure 2. It is also important to note that such coordination elongates the Si-Si bond and facilitates its dissociation.

**Scheme 3:**
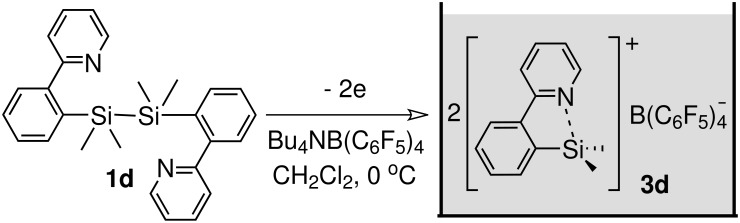
Electrochemical generation of organosilicon cation **3d**.

Preparative electrochemical oxidation of **1d** was carried out to generate and accumulate the corresponding organosilicon cation **3d** (Scheme 3). Nature of the counter anion was very important. When **1d** was oxidized in the presence of Bu_4_NBF_4_, which is a common supporting electrolyte for the "cation pool" method, fluoride was introduced on the silicon atom. Eventually, Bu_4_NB(C_6_F_5_)_4_ was found to be an appropriate supporting electrolyte to generate and accumulate the organosilicon cation **3d**.

The ^1^H NMR spectrum of the solution obtained by the electrochemical oxidation of **1d** in CH_2_Cl_2_ (containing 10% CD_2_Cl_2_) using Bu_4_NB(C_6_F_5_)_4_ at 0°C showed complete conversion of disilane **1d** to one species, *i.e*. organosilicon cation **3d**. The Si-CH_3_ groups in **3d** exhibited a signal at 0.87 ppm, whereas those in **1d** were observed at -0.05 ppm. Significant low field shift of the protons on the pyridyl ring was also observed. **3d** exhibited a ^29^Si signal at 37.7 ppm [26–28]. These results strongly suggest the generation of an electron deficient silicon species. Therefore, it is reasonable to consider that the organosilicon cation stabilized by the pyridyl coordination was generated.

**Figure 3:**
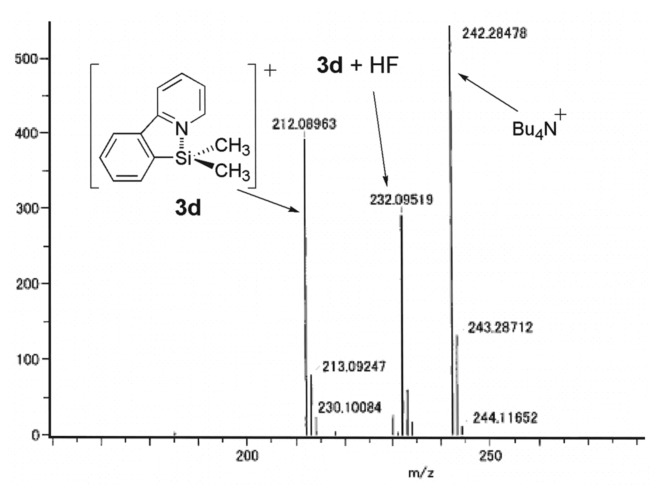
CSI-MS of organosilicon cation 3d at 0°C.

The formation of organosilicon cation **3d** was also confirmed by CSI-MS (cold-spray ionization mass spectroscopy) (spray temperature; 0°C) [29]. The parent peak was observed at *M/Z* = 212.08963 (Calcd: 212.08955) as shown in Figure 3. A complex of **3d** with HF was also observed, although the mechanism of its formation is not clear at present.

**Figure 4:**
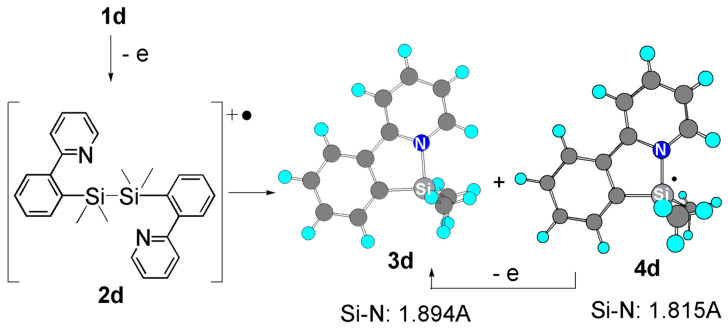
Optimized structures of organosilicon cation **3d** and silyl radical **4d** by DFT calculations (B3LYP/LANL2DZ).

The mechanism shown in Figure 4 seems to be reasonable. The initial one-electron oxidation of disilane **1d** gives radical cation **2d**. The pyridyl coordination in the radical cation facilitates the electron transfer. In the next step, the dissociation of the Si-Si bond in radical cation **2d** takes place to give organosilicon cation **3d** and silyl radical **4d**. DFT calculations indicated that the pyridyl group coordination to silicon takes place both in cation and radical. Radical **4d** seems to be easily oxidized on the surface of the electrode to give cation **3d**. Therefore, two moles of **3d** should be formed from one mole of **1d** by net two-electron oxidation.

**Scheme 4:**
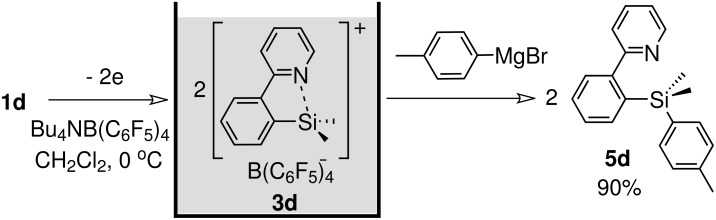
Reaction of organosilicon cation 3d with *p*-tolylmagnesium bromide.

The organosilicon cation **3d** can be trapped by *p*-tolylmagnesium bromide as a nucleophile and the corresponding product **5d** was obtained in 90% yield based on disilane **1d** (Scheme 4) [30]. The observation indicates that two moles of the cation is formed from one mole of the disilane with the consumption of two moles of electrons, being consistent with the mechanism shown in Figure 4.

Effective formation of **5d** indicates that organosilicon cation **3d** acted as a silicon centered cation. The carbon nucleophile attacked the silicon atom selectively, although a positive charge should also be delocalized on the nitrogen atom.

The present observations speak well for possibilities of the "cation pool" method in organosilicon chemistry. Donor-stabilized organosilicon cations can be effectively generated and accumulated at 0°C by the electrochemical oxidative Si-Si bond dissociation. It is also noteworthy that the presence of a donor ligand on the silicon atom facilitates the oxidation. Further work aimed at generating other organosilicon cations and exploring their stability and reactivity is currently in progress.

## Supporting Information

File 1 Supporting information. Experimental procedures, spectrum data of new compounds, details of DFT calculation, and ^1^H/^13^C NMR spectra.
