# Supplementary material for: Generation of pyridyl coordinated organosilicon cation pool by oxidative Si-Si bond dissociation
Source: Beilstein J Org Chem. 2007 Feb 8;3:7. doi: 10.1186/1860-5397-3-7 (PMC1810300; doi:10.1186/1860-5397-3-7)
Supplement: File 1 — Supporting information. Experimental procedures, spectrum data of new compounds, details of DFT calculation, and 1H/13C NMR spectra. [file Beilstein_J_Org_Chem-03-07-s001.pdf]

Supporting Information

# Generation of Pyridyl Coordinated Organosilicon Cation Pool by Oxidative Si-Si Bond Dissociation

Toshiki Nokami, Ryoji Soma, Yoshimasa Yamamoto, Toshiyuki Kamei, Kenichiro Itami, and Jun-ichi Yoshida*

*Department of Synthetic Chemistry & Biological Chemistry, Graduate School of Engineering, Kyoto University, Nishikyo-ku, Kyoto 615-8510, Japan*

**General.** $^{1}$H and $^{13}$C NMR spectra were recorded on Varian MERCURY plus-400 ($^{1}$H 400 MHz, $^{13}$C 100 MHz), JEOL ECA-600P ($^{1}$H 600 MHz, $^{13}$C 150 MHz) using the residual proton ($CDCl_2$: 5.32 ppm, $CDCl_3$: 7.26 ppm) or carbon ($CD_2Cl_2$: 53.80 ppm, $CDCl_3$: 77.00 ppm) as an internal standard. $^{29}$Si NMR spectra were recorded on Varian Gemini 2000 (60 MHz), JEOL JNM-A400 (80 MHz), JEOL JNM-A500 (100 MHz) using $Me_4Si$ as an internal standard. EI mass spectra were recorded on JMS-SX102A spectrometer. FAB mass spectra were recorded on JMS-HX110A spectrometer. CSI mass spectra were recorded on JMS-T100CSK spectrometer. Unless otherwise noted, all materials, dried diethyl ether and tetrahydrofuran were obtained from commercial suppliers and used without further purification. Dichloromethane was washed with water, distilled from $P_2O_5$, redistilled from dried $K_2CO_3$ to remove a trace amount of acid, and stored over molecular sieves 4A. Starting materials, such as tributyl(2-pyridylethyl)stannane **S1**[1] and **1c**[2] were prepared according to the reported procedure. Rotating-disk electrode voltammetry was carried out using BAS 100B and Nikko Keisoku RRDE-1 rotating disk electrode with Nikko Keisoku SC-5 controller. Measurements were carried out in 0.1 M $Bu_4NClO_4/CH_3CN$ using a glassy carbon disk working electrode, a platinum wire counter electrode, and an SCE reference electrode with sweep rate of 10 mV/s at 5000 r.p.m.

**Preparation of 1,1,2,2-Tetramethyl-1,2-bis(2-pyridylethyl)disilane (1b).**

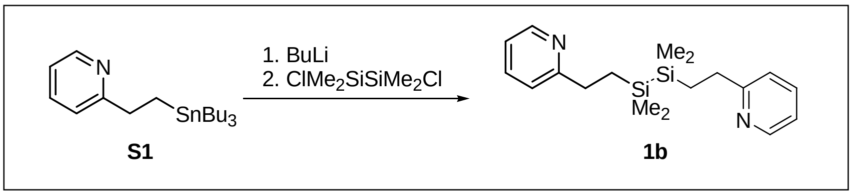


To a solution of tributyl(2-pyridylethyl)stannane **S1**[1] (15.9 g, 40.1 mmol) in THF (100 mL) was added dropwise a hexane solution of *n*-BuLi (44 mmol) at –78 °C under argon. Additional stirring (–78 °C, 30 min) of the mixture afforded a THF solution of 2-pyridylethyl lithium. To this solution, was added 1,2-dichloro-1,1,2,2-tetramethyldisilane (4.32 g, 23.1 mmol) dropwise and the mixture was stirred for 1 h at –78 °C and an additional 1 h at room temperature. The resulting mixture was treated with saturated aq $NaHCO_3$ (40 mL). After extracted with $Et_2O$ (40 mL × 3), organic phase was dried over $MgSO_4$. The solvent was removed under reduced pressure and the crude product was purified with flash chromatography (hexane / ethyl acetate 10:1, 1:1, 2:1 and 1:2) to give the title compound in 42% yield (2.79 g). $^{1}$H NMR (400 MHz, $CDCl_3$) δ 0.09 (s, 12H), 1.02-1.06 (m, 4H), 2.76-2.80 (m, 4H), 7.02 (dd, $J$ = 7.6, 4.8 Hz, 2H), 7.12 (d, $J$ = 7.6 Hz, 2H), 7.52 (dd, $J$ = 7.6, 0.8 Hz, 2H), 8.46 (dd, $J$ = 4.8, 0.8 Hz, 2H). $^{13}$C NMR (100 MHz, $CDCl_3$) δ -4.0, 15.4, 33.2, 120.6, 121.7, 136.1, 148.8, 164.1. $^{29}$Si NMR (100 MHz, $CDCl_3$) δ -17.1. HRMS (FAB) *m/z* calcd for $C_{18}H_{29}N_2Si_2$ (M+H): 329.1869, found 329.1868.

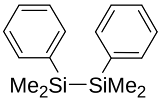


**1,1,2,2-Tetramethyl-1,2-diphenyldisilane (1c).**[2] 80 % yield from PhMgBr and 1,2-dichlorotetramethyldisilane. $^{1}$H NMR (400 MHz, $CDCl_3$) δ 0.37 (s, 12H), 7.32-7.35 (m, 6H), 7.40-7.43 (m, 4H). $^{13}$C NMR (100 MHz, $CDCl_3$) δ -3.7, 127.6, 128.3, 133.7, 138.8. $^{29}$Si NMR (60 MHz, $CDCl_3$) δ -21.8.

**Preparation of 1,1,2,2-Tetramethyl-1,2-bis[*o*-(2-pyridyl)phenyl]disilane (1d).**

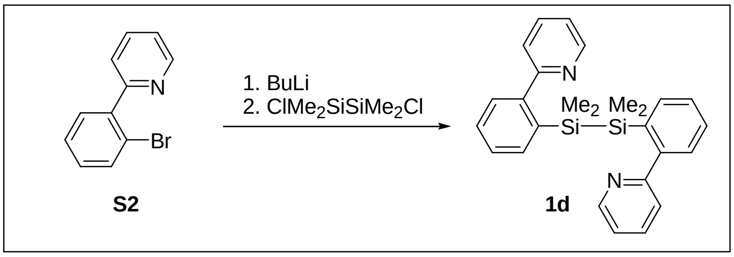


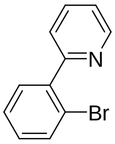

***o*-(2-Pyridyl)phenylbromide (S2).** A solution of 2-bromopyridine (2.83 g, 17.9 mmol), $Pd(PPh_3)_4$ (564 mg, 0.48 mmol), *o*-(2-bromo)phenylboronic acid (2.71 g, 12 mmol), $K_2CO_3$ (8.3 g, 60 mmol) and $H_2O$ (6.5 g, 300 mmol) in DME (15 mL) was stirred at 100 $^{o}$C (reflux) for 24 h under argon. Then, the mixture was cooled to room temperature and treated with saturated aq $NaHCO_3$ (10 mL). After extracted with $Et_2O$ (15 mL × 3), organic phase was dried over $MgSO_4$. The solvent was removed under reduced pressure and the crude product was purified by distillation (0.067 mmHg, 123 $^{o}$C) to give the title compound in 62% (1.95 g) yield. $^{1}$H NMR (400 MHz, $CDCl_3$) δ 7.23-7.31 (m, 2H), 7.40 (td, *J* = 7.6, 1.2 Hz, 1H), 7.53 (dd, *J* = 7.6, 2.0 Hz, 1H), 7.60 (dt, *J* = 8.0, 1.2 Hz, 1H), 7.67 (dt, *J* = 8.0, 1.2 Hz, 1H), 7.76 (td, *J* = 7.6, 2.0 Hz, 1H), 8.71 (dm, *J* = 4.8 Hz, 1H). $^{13}$C NMR (100 MHz, $CDCl_3$) δ 121.6, 122.2, 124.5, 127.3, 129.5, 131.2, 133.0, 135.5, 141.0, 149.1, 158.0. HRMS (EI) *m/z* calcd for $C_{11}H_8NBr$ (M+): 232.9840, found 232.9842.

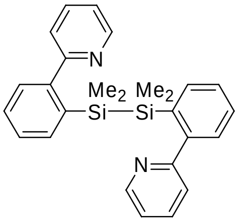

**1,1,2,2-Tetramethyl-1,2-bis[*o*-(2-pyridyl)phenyl]disilane (1d).** To a solution of *o*-(2-pyridyl)phenylbromide **S2** (1.32 g, 5.6 mmol) in $Et_2O$ (10 mL) was added dropwise a *n*-hexane solution of *n*-BuLi (5.7 mmol) at –78 $^{o}$C under argon. Additional stirring (–78 $^{o}$C, 90 min) of the mixture afforded a $Et_2O$ solution of *o*-(2-pyridyl)phenyl lithium. To this solution, was added 1,2-dichloro-1,1,2,2-tetramethyldisilane (505.5 mg, 2.7 mmol) dropwise and stirred at –78 $^{o}$C to room temperature for 20 h. The resulting mixture was treated with saturated aq $NaHCO_3$ (10 mL). After extracted with $Et_2O$ (10 mL × 3), organic phase was dried over $MgSO_4$. The solvent was removed under reduced pressure and the crude product was purified with flash chromatography (hexane / ethyl acetate 5:1) to give the title compound in 85% yield (974.1 mg). $^{1}$H NMR (400 MHz, $CDCl_3$) δ 0.07 (s, 12H), 7.08 (ddd, *J* = 6.4, 4.8, 1.6 Hz, 2H), 7.39 (td, *J* = 7.2, 1.6 Hz, 2H), 7.46 (td, *J* = 7.2, 1.6 Hz, 2H), 7.60-7.66 (m, 6H), 7.71 (dd, *J* = 7.8, 1.6 Hz, 2H), 8.18 (dt, *J* = 4.8, 0.8 Hz, 2H). $^{1}$H NMR (600 MHz, $CH_2Cl_2$ containing 10% $CD_2Cl_2$, 0 $^{o}$C) δ -0.05 (s, 12H), 7.06 (ddd, *J* = 7.5, 6.3, 1.2 Hz, 2H), 7.34 (td, *J* = 7.5, 1.2 Hz, 2H), 7.42 (td, *J* = 7.5, 1.7 Hz, 2H), 7.57 (d, *J* = 8.0 Hz, 2H), 7.61-7.64 (m, 6H), 8.08 (d, *J* = 5.2 Hz, 2H). $^{13}$C NMR (100 MHz, $CDCl_3$) δ 0.2, 121.3, 122.0, 127.3, 127.8, 128.0, 135.9, 136.2, 141.1, 145.2, 148.0, 160.1. $^{29}$Si NMR (80 MHz, $CDCl_3$) δ -21.0. HRMS (FAB) *m/z* calcd for $C_{26}H_{29}N_2Si_2$ (M+H): 425.1869, found 425.1874.

**Preparation of Dimethyl[*o*-(2-pyridyl)phenyl]silylium ion (3d).**

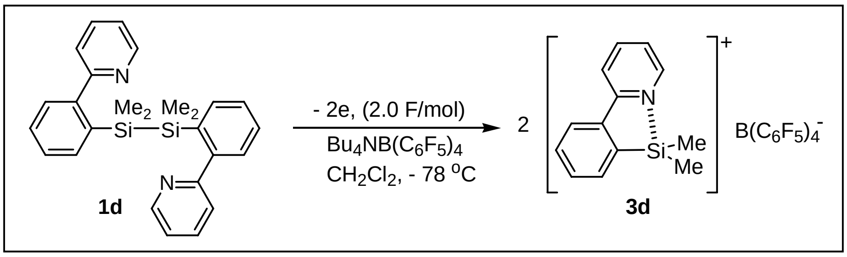


The anodic oxidation was carried out in an H-type divided cell equipped with a 4G glass filter (separator), a carbon felt anode (Nippon Carbon JF-20-P7, ca. 160 mg, dried at 250 °C/1 mmHg for 2.5 h before use) and a platinum plate cathode (10 mm x 10 mm). In the anodic chamber was placed a solution of **1d** (84.9 mg, 0.2 mmol) in 0.1 M $Bu_4NB(C_6F_5)_4/CH_2Cl_2$ (10% $CD_2Cl_2$) (4 mL). In the cathodic chamber was placed 0.1 M $Bu_4NB(C_6F_5)_4/CH_2Cl_2$ (10% $CD_2Cl_2$) (4 mL). The constant current electrolysis (5 mA) was carried out at 0 °C with magnetic stirring until 2 F/mol of electricity was consumed. The resulting solution in the anodic chamber was analyzed by NMR. $^1H$ NMR (600 MHz, $CH_2Cl_2$ containing 10% $CD_2Cl_2$, 0 °C) δ 0.87 (s, 6H), 7.73 (ddd, $J$ = 7.5, 6.6, 0.6 Hz, 1H), 7.78 (ddd, $J$ = 7.8, 7.5, 0.6 Hz, 1H), 7.83 (ddd, $J$ = 6.6, 6.0, 0.6 Hz, 1H), 7.92 (d, $J$ = 6.6 Hz, 1H), 8.11 (d, $J$ = 7.8 Hz, 1H), 8.36 (d, $J$ = 7.8 Hz, 1H), 8.50 (ddd, $J$ = 7.8, 6.0, 0.6 Hz, 1H), 8.52 (d, $J$ = 6.0 Hz, 1H). $^{13}C$ NMR (150 MHz, $CD_2Cl_2$, 0 °C) δ -2.3, 121.8, 125.1, 126.4, 132.1, 133.6, 133.9, 134.1, 137.1, 143.6, 148.1, 157.3. $^{29}Si$ NMR (80 MHz, $CD_2Cl_2$) δ 37.7. HRMS (CSI) *m/z* calcd for $C_{13}H_{14}NSi$ ($M^+$): 212.08955, found 212.08963.

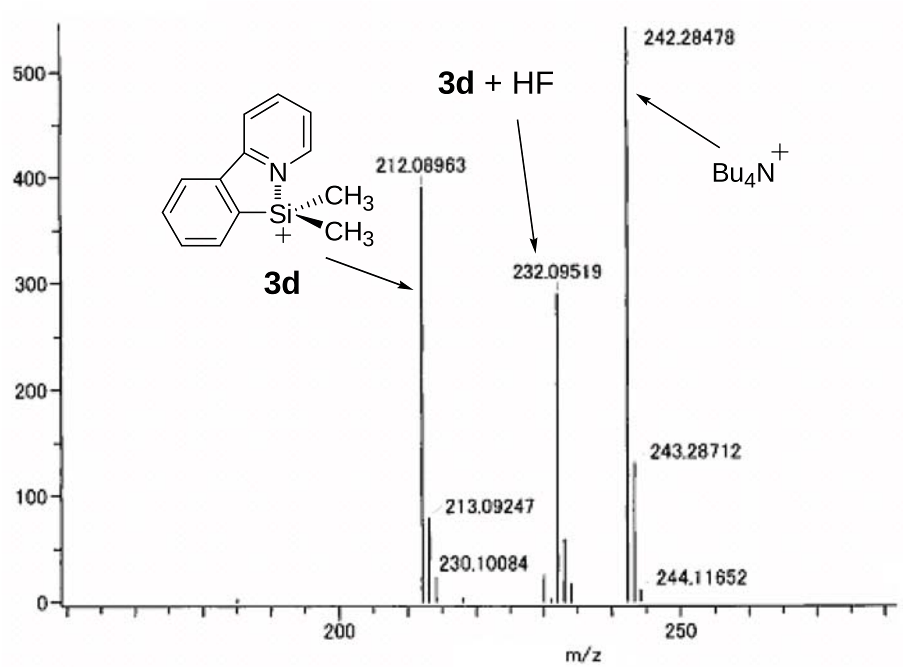


CSI-MS of **3d** at 0 °C.

**Preparation of Dimethyl[*o*-(2-pyridyl)phenyl]-*p*-tolylsilane (5d).**

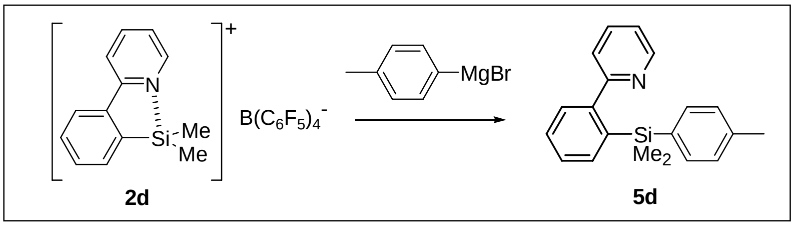


The electrochemical oxidation of **1d** (61.1 mg, 0.14 mmol) was carried out in 0.1 M $Bu_4NB(C_6F_5)_4/CH_2Cl_2$ as described above. A $Et_2O$ solution of *p*-tolylmagnesium bromide (0.375 mmol) was added to the anodic chamber and the mixture was stirred at 0 °C for 1 h. The solution in the anodic chamber was treated with saturated aq

$NaHCO_3$ (4 mL). After extracted with $CHCl_3$ (4 mL × 3), organic phase was dried over $MgSO_4$. Removal of the solvent under reduced pressure and the residue was quickly filtered through a short column (2 x 3 cm) of silica gel to remove $Bu_4NB(C_6F_5)_4$. The silica gel was washed with diethyl ether (50 mL). The solvent was removed from the combined filtrate under reduced pressure. $^1$H NMR analysis of the crude product using 1,1,2,2-tetrachloroethane as internal standard indicated that the title compound was formed in 90% yield. The product was purified with flash chromatography (hexane / ethyl acetate 10:1) to give the title compound in 68% (58.7 mg) yield. $^1$H NMR (400 MHz, $CDCl_3$) δ 0.32 (s, 6H), 2.33 (s, 3H), 7.09 (d, *J* = 7.6 Hz, 2H), 7.16 (ddd, *J* = 7.6, 4.8, 1.2 Hz, 1H), 7.32-7.38 (m, 4H), 7.44 (td, *J* = 7.6, 1.6 Hz, 1H), 7.50 (dd, *J* = 7.6, 1.6 Hz, 1H), 7.59 (td, *J* = 7.6, 1.6 Hz, 1H), 7.62 (dd, *J* = 7.6, 1.6 Hz, 1H), 8.51 (dd, *J* = 4.8, 1.2 Hz, 1H). $^{13}$C NMR (150 MHz, $CDCl_3$) δ -0.4, 21.4, 121.9, 122.9, 127.4, 128.3, 128.6, 129.0, 133.8, 136.2, 136.7, 137.0, 137.5, 138.0, 147.0, 148.2, 160.6. HRMS (FAB) *m/z* calcd for $C_{20}H_{25}N_2Si_1$ (M+H): 304.1522, found 304.1524.

**DFT Calculations.**

The DFT calculations were carried out at B3LYP/LANL2DZ level using the Gaussian 2003W, Revision-B.05.[7] Geometries were fully optimized. The local minima were verified to have no negative eigenvalue by the vibration analysis. Cartesian coordinates and energies of computationally characterized species are as follows:

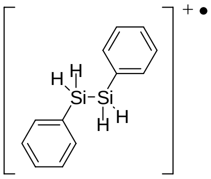

| Center Number | Atomic Number | Atomic Type | Coordinates (Angstroms) X | Y | Z |
|---|---|---|---|---|---|
| 1 | 14 | 0 | -1.113746 | 0.290215 | 0.466800 |
| 2 | 14 | 0 | 1.121377 | -0.288551 | -0.452600 |
| 3 | 6 | 0 | -1.879117 | -1.335575 | 0.955795 |
| 4 | 6 | 0 | 1.886531 | 1.337262 | -0.941782 |
| 5 | 6 | 0 | -1.692778 | -1.864633 | 2.273778 |
| 6 | 6 | 0 | -2.313688 | -3.058227 | 2.656262 |
| 7 | 6 | 0 | -3.113040 | -3.768108 | 1.726085 |
| 8 | 6 | 0 | -3.292910 | -3.272614 | 0.410696 |
| 9 | 6 | 0 | -2.673616 | -2.079375 | 0.024521 |
| 10 | 6 | 0 | 2.680729 | 2.081401 | -0.010519 |
| 11 | 6 | 0 | 3.299812 | 3.274707 | -0.396819 |
| 12 | 6 | 0 | 3.120042 | 3.769929 | -1.712328 |
| 13 | 6 | 0 | 2.321003 | 3.059701 | -2.642511 |
| 14 | 6 | 0 | 1.700301 | 1.866047 | -2.259898 |
| 15 | 1 | 0 | -1.819359 | 0.956370 | -0.647582 |
| 16 | 1 | 0 | -0.831280 | 1.172546 | 1.617980 |
| 17 | 1 | 0 | 0.839148 | -1.171145 | -1.603631 |
| 18 | 1 | 0 | 1.826989 | -0.954356 | 0.661994 |
| 19 | 1 | 0 | -1.082498 | -1.327845 | 2.996394 |
| 20 | 1 | 0 | -2.185911 | -3.441015 | 3.664436 |
| 21 | 1 | 0 | -3.585681 | -4.701349 | 2.021297 |
| 22 | 1 | 0 | -3.912239 | -3.818965 | -0.294428 |
| 23 | 1 | 0 | -2.819637 | -1.708164 | -0.987230 |
| 24 | 1 | 0 | 2.826677 | 1.710399 | 1.001319 |
| 25 | 1 | 0 | 3.918909 | 3.821329 | 0.308300 |
| 26 | 1 | 0 | 3.592527 | 4.703224 | -2.007620 |
| 27 | 1 | 0 | 2.193309 | 3.442274 | -3.650777 |
| 28 | 1 | 0 | 1.090257 | 1.328991 | -2.982516 |

Zero-point correction= 0.216454 (Hartree/Particle)
Thermal correction to Energy= 0.230809
Thermal correction to Enthalpy= 0.231753
Thermal correction to Gibbs Free Energy= 0.171505
Sum of electronic and zero-point Energies= -472.886084
Sum of electronic and thermal Energies= -472.871728
Sum of electronic and thermal Enthalpies= -472.870784
Sum of electronic and thermal Free Energies= -472.931033

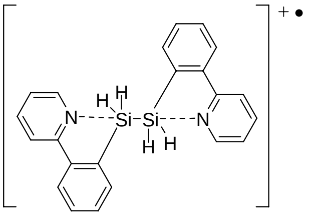


| Center Number | Atomic Number | Atomic Type | Coordinates (Angstroms) X | Y | Z |
|---|---|---|---|---|---|
| 1 | 14 | 0 | 1.537159 | 0.417148 | 0.005581 |
| 2 | 14 | 0 | -1.425340 | -0.245552 | 0.215020 |
| 3 | 6 | 0 | 1.382309 | 2.279138 | -0.365278 |
| 4 | 6 | 0 | -1.270497 | -2.107509 | 0.586037 |
| 5 | 6 | 0 | 0.198774 | 3.028793 | -0.485673 |
| 6 | 6 | 0 | 0.244325 | 4.409975 | -0.764854 |
| 7 | 6 | 0 | 1.482306 | 5.059717 | -0.927531 |
| 8 | 6 | 0 | 2.677295 | 4.331285 | -0.811687 |
| 9 | 6 | 0 | 2.629093 | 2.950143 | -0.532443 |
| 10 | 6 | 0 | 3.831088 | 2.104672 | -0.393174 |
| 11 | 6 | 0 | 5.174385 | 2.515320 | -0.510430 |
| 12 | 6 | 0 | 6.198855 | 1.571635 | -0.346801 |
| 13 | 6 | 0 | 5.876094 | 0.226158 | -0.067598 |
| 14 | 6 | 0 | 4.527336 | -0.126844 | 0.038197 |
| 15 | 7 | 0 | 3.543536 | 0.793047 | -0.121697 |
| 16 | 6 | 0 | -2.517287 | -2.778531 | 0.753094 |
| 17 | 6 | 0 | -2.565489 | -4.159638 | 1.032515 |
| 18 | 6 | 0 | -1.370495 | -4.888019 | 1.148643 |
| 19 | 6 | 0 | -0.132510 | -4.238258 | 0.986085 |
| 20 | 6 | 0 | -0.086961 | -2.857110 | 0.706732 |
| 21 | 6 | 0 | -3.719284 | -1.933103 | 0.613591 |
| 22 | 6 | 0 | -5.062590 | -2.343787 | 0.730645 |
| 23 | 6 | 0 | -6.087058 | -1.400133 | 0.566831 |
| 24 | 6 | 0 | -5.764291 | -0.054656 | 0.287637 |
| 25 | 6 | 0 | -4.415525 | 0.298379 | 0.182037 |
| 26 | 7 | 0 | -3.431726 | -0.621483 | 0.342103 |
| 27 | 1 | 0 | 1.612165 | -0.595367 | -1.081475 |
| 28 | 1 | 0 | 1.675727 | -0.096813 | 1.394517 |
| 29 | 1 | 0 | -1.563894 | 0.268409 | -1.173920 |
| 30 | 1 | 0 | -1.500414 | 0.766957 | 1.302077 |
| 31 | 1 | 0 | -0.768119 | 2.552506 | -0.364890 |
| 32 | 1 | 0 | -0.679296 | 4.975427 | -0.854869 |
| 33 | 1 | 0 | 1.514498 | 6.124127 | -1.142608 |
| 34 | 1 | 0 | 3.627823 | 4.841930 | -0.938944 |
| 35 | 1 | 0 | 5.411910 | 3.550744 | -0.725022 |
| 36 | 1 | 0 | 7.237696 | 1.875787 | -0.434782 |
| 37 | 1 | 0 | 6.647163 | -0.524659 | 0.063718 |
| 38 | 1 | 0 | 4.217030 | -1.145129 | 0.251184 |
| 39 | 1 | 0 | -3.516020 | -4.670293 | 1.159709 |
| 40 | 1 | 0 | -1.402691 | -5.952399 | 1.363859 |
| 41 | 1 | 0 | 0.791118 | -4.803663 | 1.076335 |
| 42 | 1 | 0 | 0.879939 | -2.380807 | 0.586066 |
| 43 | 1 | 0 | -5.300120 | -3.379213 | 0.945217 |
| 44 | 1 | 0 | -7.125906 | -1.704312 | 0.654653 |
| 45 | 1 | 0 | -6.535359 | 0.696138 | 0.156181 |
| 46 | 1 | 0 | -4.105214 | 1.316660 | -0.030960 |

Zero-point correction= 0.358910 (Hartree/Particle)
Thermal correction to Energy= 0.381025
Thermal correction to Enthalpy= 0.381969
Thermal correction to Gibbs Free Energy= 0.304229
Sum of electronic and zero-point Energies= -966.915950
Sum of electronic and thermal Energies= -966.893836
Sum of electronic and thermal Enthalpies= -966.892892
Sum of electronic and thermal Free Energies= -966.970631

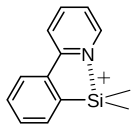

**3d**

| Center Number | Atomic Number | Atomic Type | Coordinates (Angstroms) X | Y | Z |
|---|---|---|---|---|---|
| 1 | 6 | 0 | 0.191409 | -0.429732 | -2.762887 |
| 2 | 6 | 0 | 0.304808 | 0.726339 | -3.565589 |
| 3 | 6 | 0 | 0.317469 | 2.005770 | -2.975299 |
| 4 | 6 | 0 | 0.217309 | 2.145601 | -1.580555 |
| 5 | 6 | 0 | 0.104064 | 0.991354 | -0.779149 |
| 6 | 6 | 0 | 0.090418 | -0.308140 | -1.368130 |
| 7 | 6 | 0 | -0.007472 | 1.005604 | 0.691084 |
| 8 | 14 | 0 | -0.069911 | -1.631294 | -0.046051 |
| 9 | 6 | 0 | -0.021040 | 2.133923 | 1.532824 |
| 10 | 6 | 0 | -0.134440 | 1.967063 | 2.920003 |
| 11 | 6 | 0 | -0.234741 | 0.668852 | 3.469991 |
| 12 | 6 | 0 | -0.218038 | -0.425427 | 2.607247 |
| 13 | 7 | 0 | -0.107313 | -0.256338 | 1.256309 |
| 14 | 6 | 0 | 1.442324 | -2.694580 | 0.283541 |
| 15 | 6 | 0 | -1.703478 | -2.552940 | 0.044016 |
| 16 | 1 | 0 | 0.183828 | -1.406324 | -3.240528 |
| 17 | 1 | 0 | 0.382798 | 0.629458 | -4.644766 |
| 18 | 1 | 0 | 0.405114 | 2.888937 | -3.601363 |
| 19 | 1 | 0 | 0.228583 | 3.138941 | -1.140659 |
| 20 | 1 | 0 | 0.056268 | 3.126043 | 1.103978 |
| 21 | 1 | 0 | -0.145238 | 2.835337 | 3.572012 |
| 22 | 1 | 0 | -0.323358 | 0.512750 | 4.538774 |
| 23 | 1 | 0 | -0.291760 | -1.443485 | 2.974430 |
| 24 | 1 | 0 | 2.356712 | -2.093306 | 0.337400 |
| 25 | 1 | 0 | 1.573060 | -3.424744 | -0.526376 |
| 26 | 1 | 0 | 1.344065 | -3.264344 | 1.216265 |
| 27 | 1 | 0 | -1.798443 | -3.123119 | 0.976826 |
| 28 | 1 | 0 | -1.775139 | -3.273775 | -0.781499 |
| 29 | 1 | 0 | -2.558105 | -1.872020 | -0.036529 |

Zero-point correction= 0.239061 (Hartree/Particle)
Thermal correction to Energy= 0.252719
Thermal correction to Enthalpy= 0.253663
Thermal correction to Gibbs Free Energy= 0.199003
Sum of electronic and zero-point Energies= -561.976619
Sum of electronic and thermal Energies= -561.962961
Sum of electronic and thermal Enthalpies= -561.962017
Sum of electronic and thermal Free Energies= -562.016678

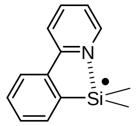

**4d**

| Center Number | Atomic Number | Atomic Type | Coordinates (Angstroms) X | Y | Z |
|---|---|---|---|---|---|
| 1 | 6 | 0 | 0.190580 | -0.432942 | -2.753313 |
| 2 | 6 | 0 | 0.304643 | 0.703271 | -3.578021 |
| 3 | 6 | 0 | 0.318117 | 1.993586 | -2.993413 |
| 4 | 6 | 0 | 0.219501 | 2.153943 | -1.606264 |
| 5 | 6 | 0 | 0.104092 | 1.014230 | -0.765986 |
| 6 | 6 | 0 | 0.090047 | -0.298339 | -1.356233 |
| 7 | 6 | 0 | -0.005430 | 1.036997 | 0.682933 |
| 8 | 14 | 0 | -0.070808 | -1.589228 | -0.009463 |
| 9 | 6 | 0 | -0.020859 | 2.147594 | 1.540335 |

| | | | | | |
|---|---|---|---|---|---|
| 10 | 6 | 0 | -0.133708 | 1.998418 | 2.931417 |
| 11 | 6 | 0 | -0.235194 | 0.668850 | 3.477221 |
| 12 | 6 | 0 | -0.220176 | -0.421209 | 2.637314 |
| 13 | 7 | 0 | -0.108114 | -0.282790 | 1.250203 |
| 14 | 6 | 0 | 1.409691 | -2.740062 | 0.248736 |
| 15 | 6 | 0 | -1.670138 | -2.601438 | 0.013750 |
| 16 | 1 | 0 | 0.181081 | -1.421027 | -3.211720 |
| 17 | 1 | 0 | 0.381995 | 0.593087 | -4.656853 |
| 18 | 1 | 0 | 0.406248 | 2.871070 | -3.630867 |
| 19 | 1 | 0 | 0.231761 | 3.153320 | -1.177396 |
| 20 | 1 | 0 | 0.057006 | 3.140550 | 1.105551 |
| 21 | 1 | 0 | -0.144559 | 2.864985 | 3.584862 |
| 22 | 1 | 0 | -0.323911 | 0.513048 | 4.548095 |
| 23 | 1 | 0 | -0.294799 | -1.436478 | 3.016431 |
| 24 | 1 | 0 | 2.343449 | -2.167975 | 0.299204 |
| 25 | 1 | 0 | 1.493562 | -3.453883 | -0.582608 |
| 26 | 1 | 0 | 1.311396 | -3.322695 | 1.174838 |
| 27 | 1 | 0 | -1.765544 | -3.184351 | 0.939978 |
| 28 | 1 | 0 | -1.690676 | -3.310424 | -0.825670 |
| 29 | 1 | 0 | -2.545903 | -1.947886 | -0.073653 |

---------------------------------------------------------------------

Zero-point correction= 0.235103 (Hartree/Particle)
Thermal correction to Energy= 0.249091
Thermal correction to Enthalpy= 0.250035
Thermal correction to Gibbs Free Energy= 0.194115
Sum of electronic and zero-point Energies= -562.152616
Sum of electronic and thermal Energies= -562.138629
Sum of electronic and thermal Enthalpies= -562.137684
Sum of electronic and thermal Free Energies= -562.193604

## References


(1) Yoshida, J.; Izawa, M. *J. Am. Chem. Soc.* **1997**, *119*, 9361.
(2) Kunai, A.; Kawakami, T.; Toyoda, E.; Ishikawa, M. *Organometallics* **1991**, *10*, 893.
(3) Li, L-S.; Das, S.; Sinha, S. C. *Org. Lett.* **2004**, *6*, 127.
(4) Chen, S-L.; Ji, S-J.; Loh, T-P. *Tetrahedron Lett.* **2004**, *45*, 375.
(5) Maruoka, K.; Hashimoto, S.; Kitagawa, Y.; Yamamoto, H.; Nozaki, H. *Bull. Chem. Soc. Jpn.* **1980**, *53*, 3301.
(6) Gnaneshwar, R.; Wadgaonkar, P. P.; Sivaram, S. *Tetrahedron Lett.* **2003**, *44*, 6047.
(7) Gaussian 03, Revision B.05, Frisch, M. J.; Trucks, G. W.; Schlegel, H. B.; Scuseria, G. E.; Robb, M. A.; Cheeseman, J. R.; Montgomery, Jr., J. A.; Vreven, T.; Kudin, K. N.; Burant, J. C.; Millam, J. M.; Iyengar, S. S.; Tomasi, J.; Barone, V.; Mennucci, B.; Cossi, M.; Scalmani, G.; Rega, N.; Petersson, G. A.; Nakatsuji, H.; Hada, M.; Ehara, M.;Toyota, K.; Fukuda, R.; Hasegawa, J.; Ishida, M.; Nakajima, T.; Honda, Y.; Kitao, O.; Nakai, H.; Klene, M.; Li, X.; Knox, J. E.; Hratchian, H. P.; Cross, J. B.; Adamo, C.; Jaramillo, J.; Gomperts, R.; Stratmann, R. E.; Yazyev, O.; Austin, A. J.; Cammi, R.; Pomelli, C.; Ochterski, J. W.; Ayala, P. Y.; Morokuma, K.; Voth, G. A.; Salvador, P.; Dannenberg, J. J.; Zakrzewski, V. G.; Dapprich, S.; Daniels, A. D.; Strain, M. C.; Farkas, O.; Malick, D. K.; Rabuck, A. D.; Raghavachari, K.; Foresman, J. B.; Ortiz, J. V.; Cui, Q.; Baboul, A. G.; Clifford, S.; Cioslowski, J.; Stefanov, B. B.; Liu, G.; Liashenko, A.; Piskorz, P.; Komaromi, I.; Martin, R. L.; Fox, D. J.; Keith, T.; Al-Laham, M. A.; Peng, C. Y.; Nanayakkara, A.; Challacombe, M.; Gill, P. M. W.; Johnson, B.; Chen, W.; Wong, M. W.; Gonzalez, C.; Pople, J. A.; Gaussian, Inc., Pittsburgh PA, 2003.

$Me_3Si—SiMe_3$

**1a**

$^{29}Si$ NMR

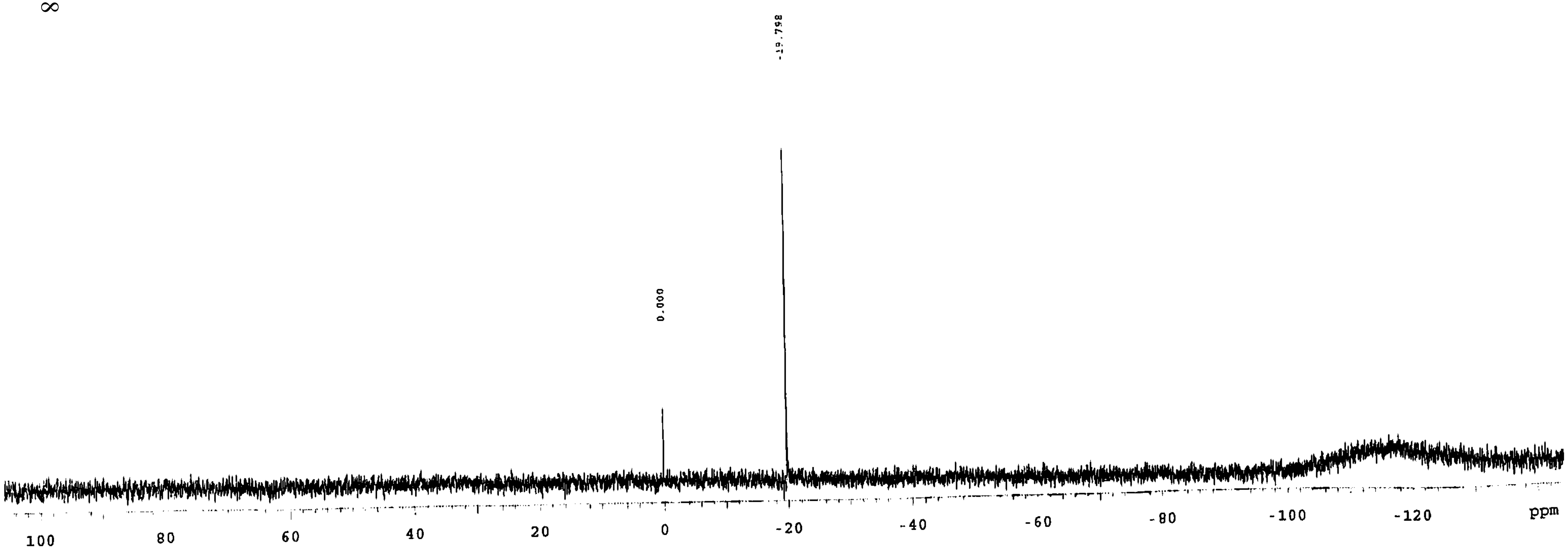

¹H NMR

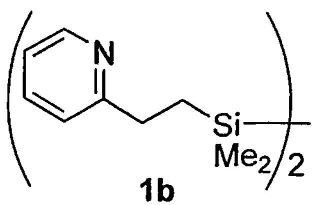

N
Si
Me2
2


**1b**

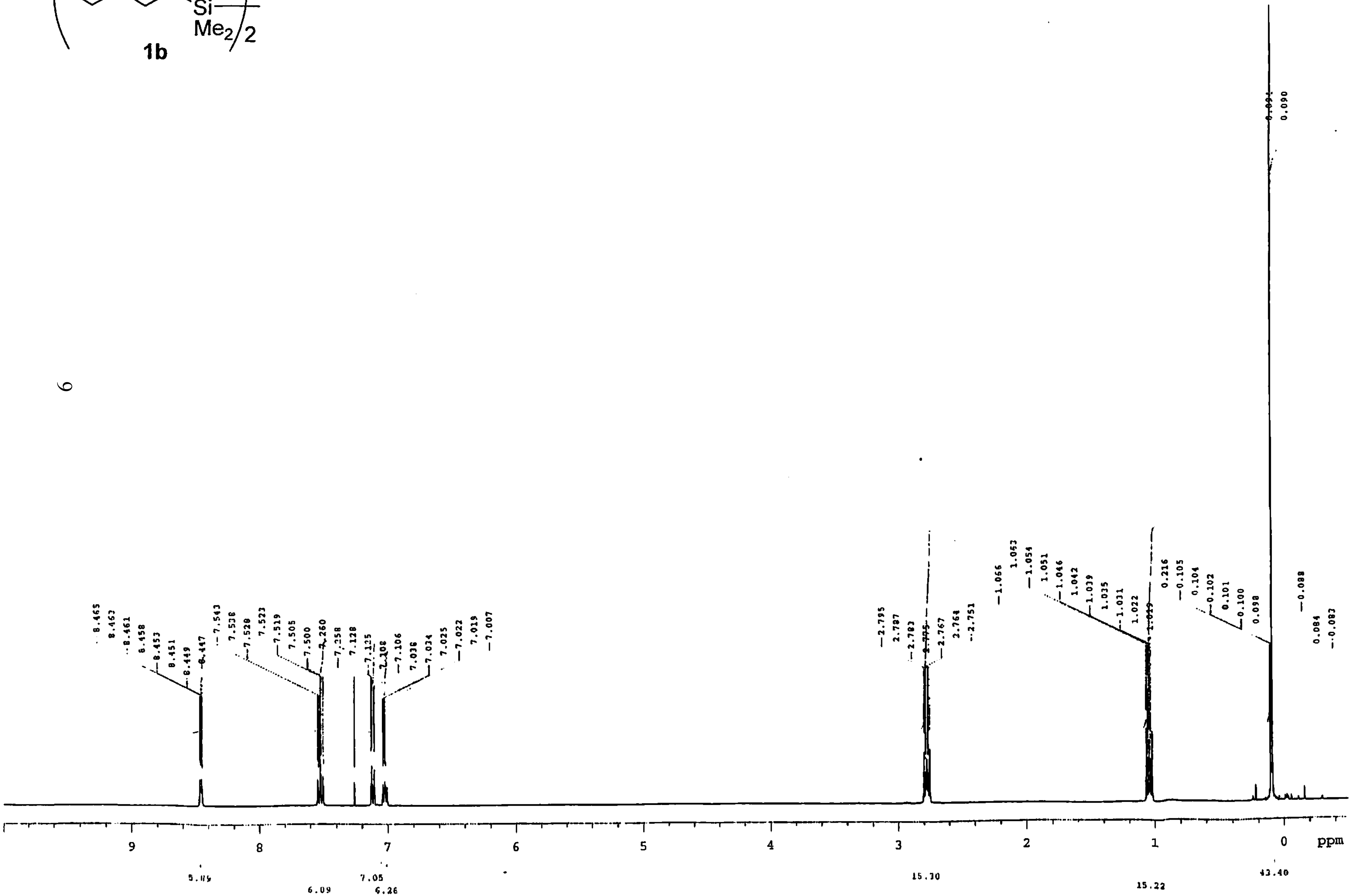

8.465
8.463
8.461
8.458
8.453
8.451
8.449
8.447
7.543
7.538
7.528
7.523
7.519
7.505
7.500
7.260
7.258
7.128
7.125
7.108
7.106
7.036
7.034
7.025
7.022
7.019
7.007
2.795
2.787
2.783
2.775
2.767
2.764
2.751
1.066
1.063
1.054
1.051
1.046
1.042
1.039
1.035
1.031
1.022
1.019
0.216
0.105
0.104
0.102
0.101
0.100
0.098
0.091
0.090
0.088
0.084
0.083
9
8
7
6
5
4
3
2
1
0
ppm
5.09
6.09
7.05
6.26
15.30
15.22
43.40

## $^{13}C$ NMR

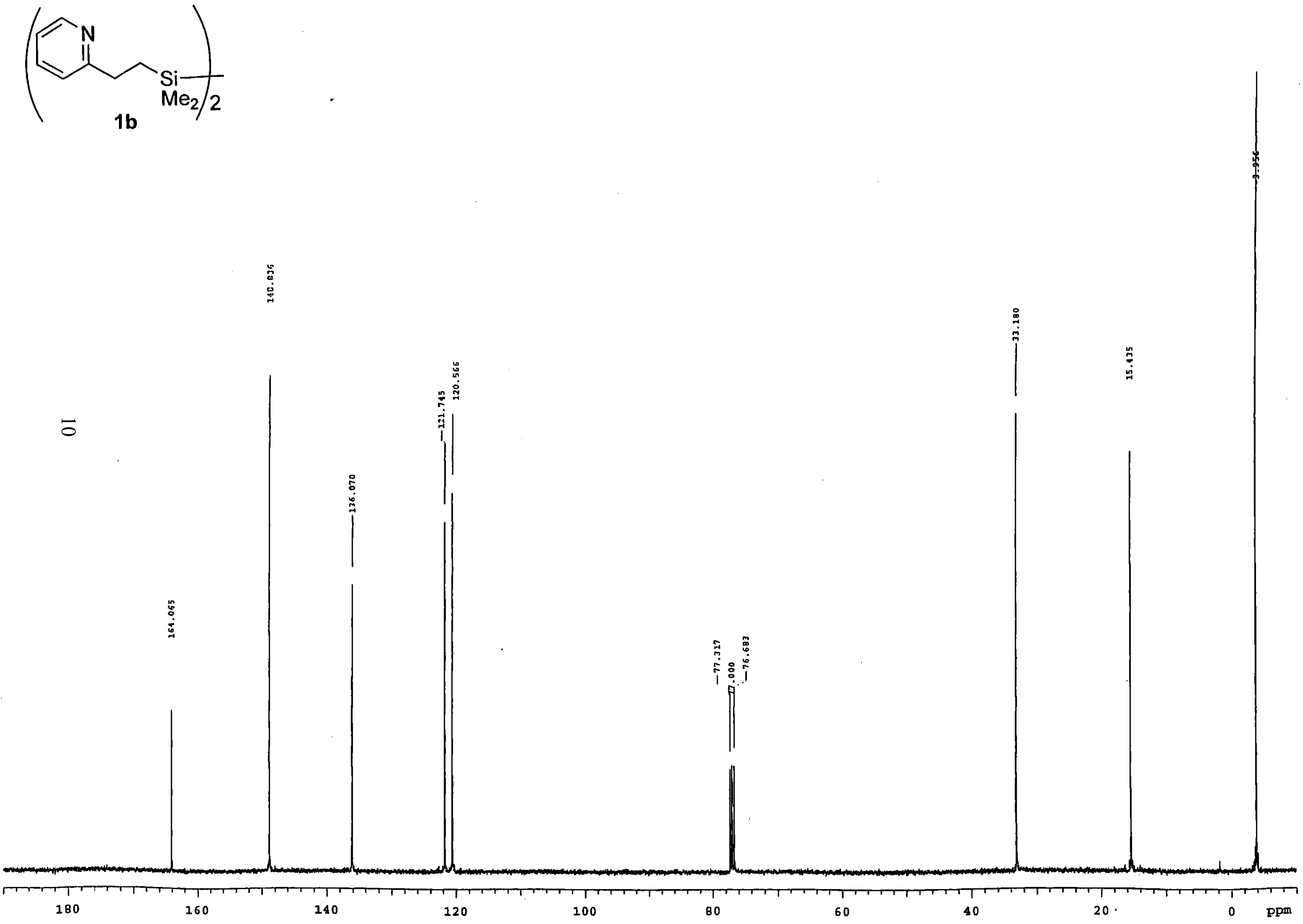

1b
164.065
148.836
136.070
121.745
120.566
77.317
77.000
76.683
33.180
15.435
3.956
180
160
140
120
100
80
60
40
20
0
ppm

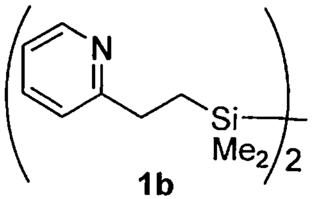

**1b**

$^{29}Si$ NMR

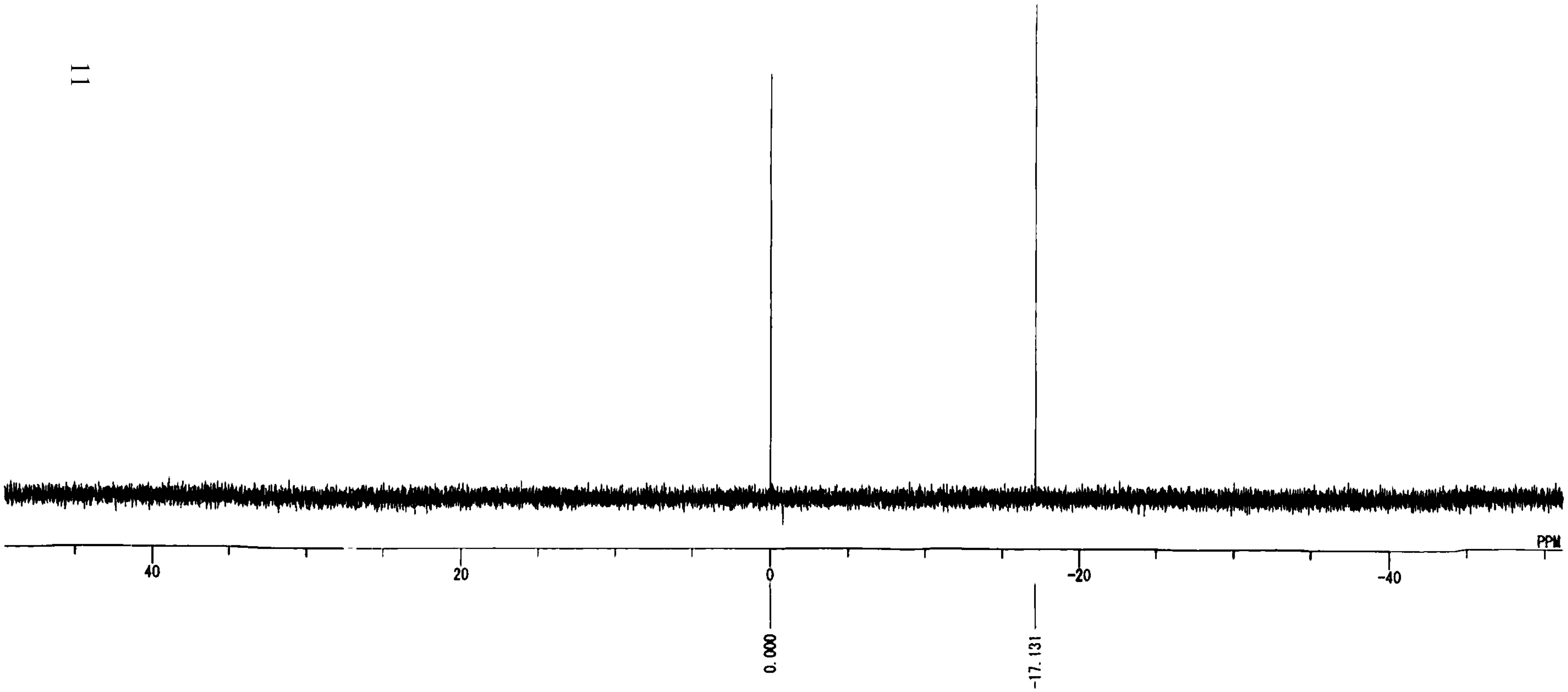

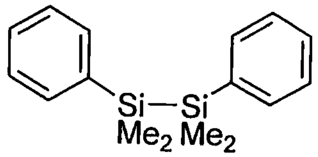


**1c**

$^1H$ NMR

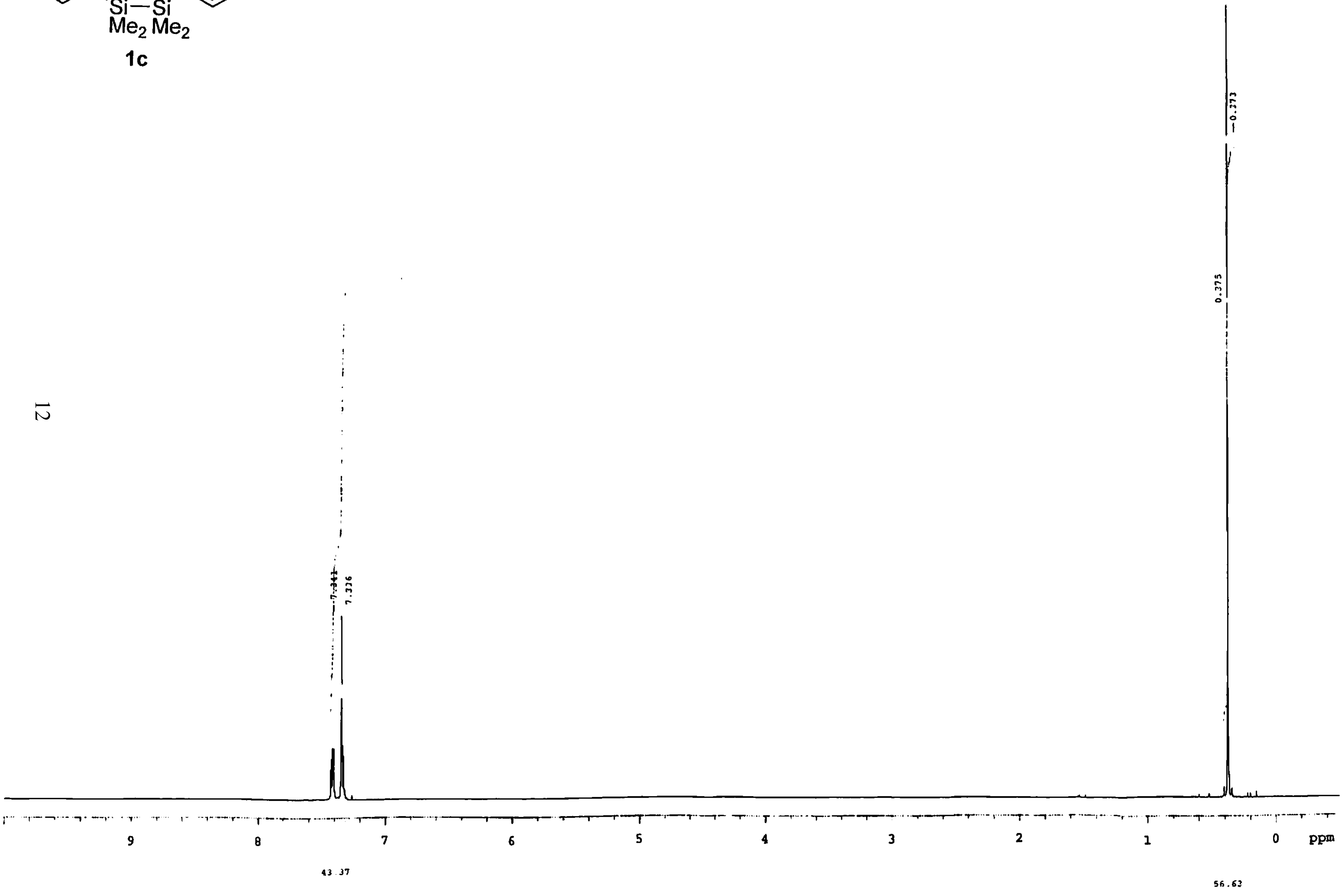

## $^{13}C$ NMR

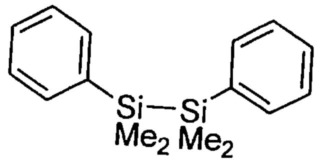

**1c**

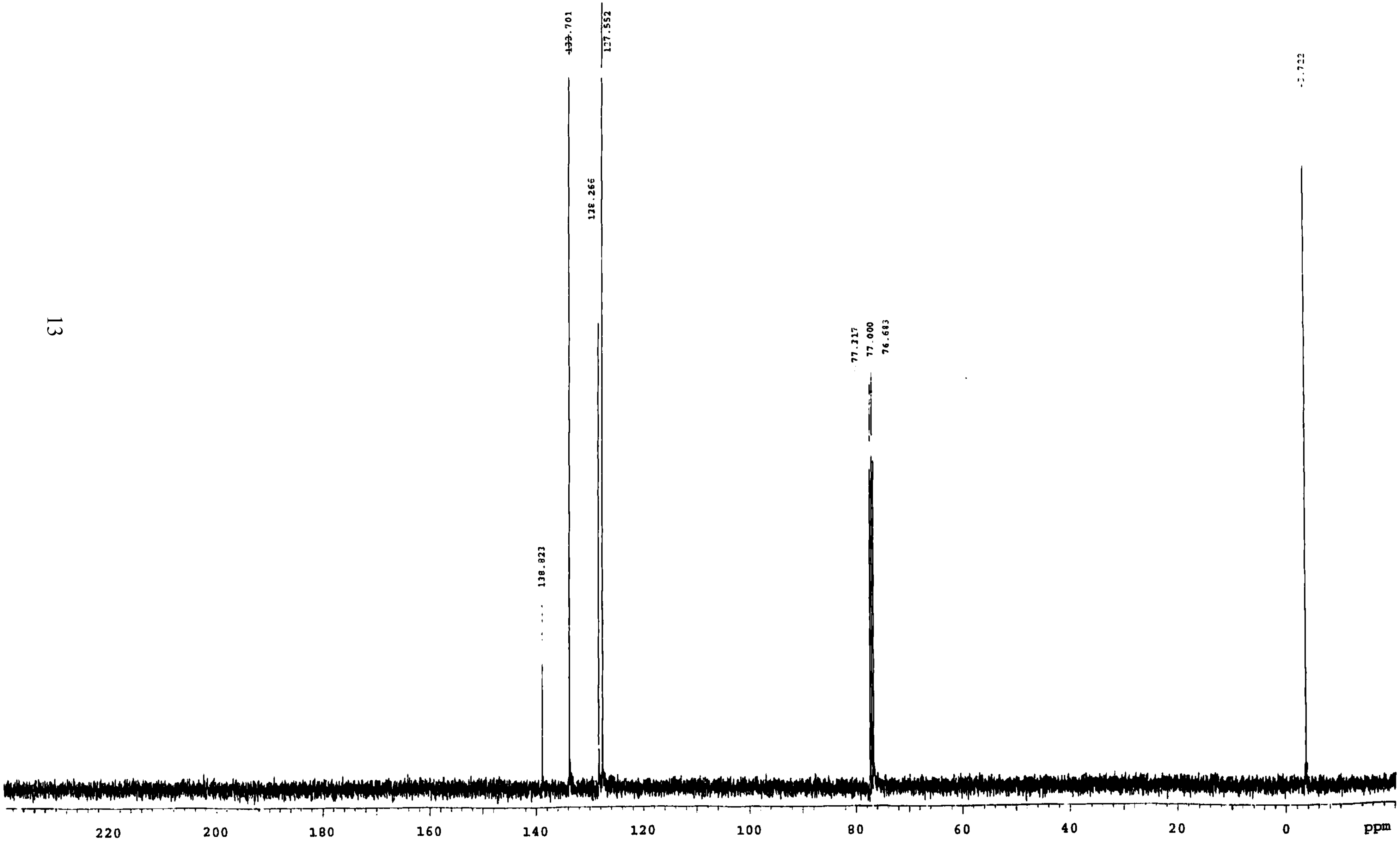
133.701
127.552
128.266
138.823
77.317
77.000
76.683
220
200
180
160
140
120
100
80
60
40
20
0
ppm

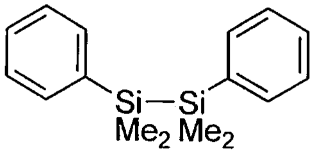


**1c**

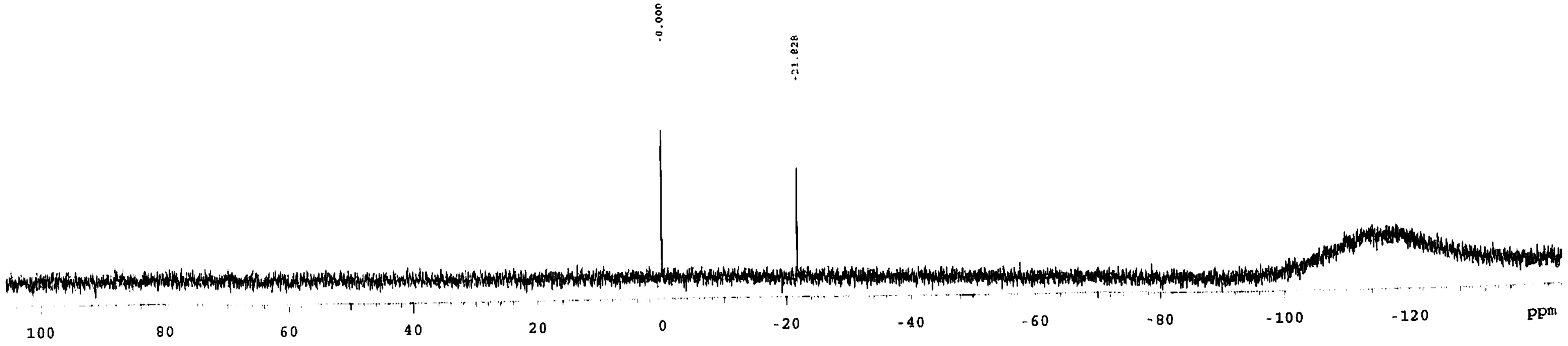


$^{29}$Si NMR

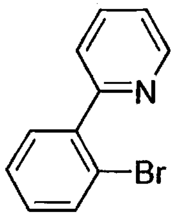
N
Br

$^1H$ NMR

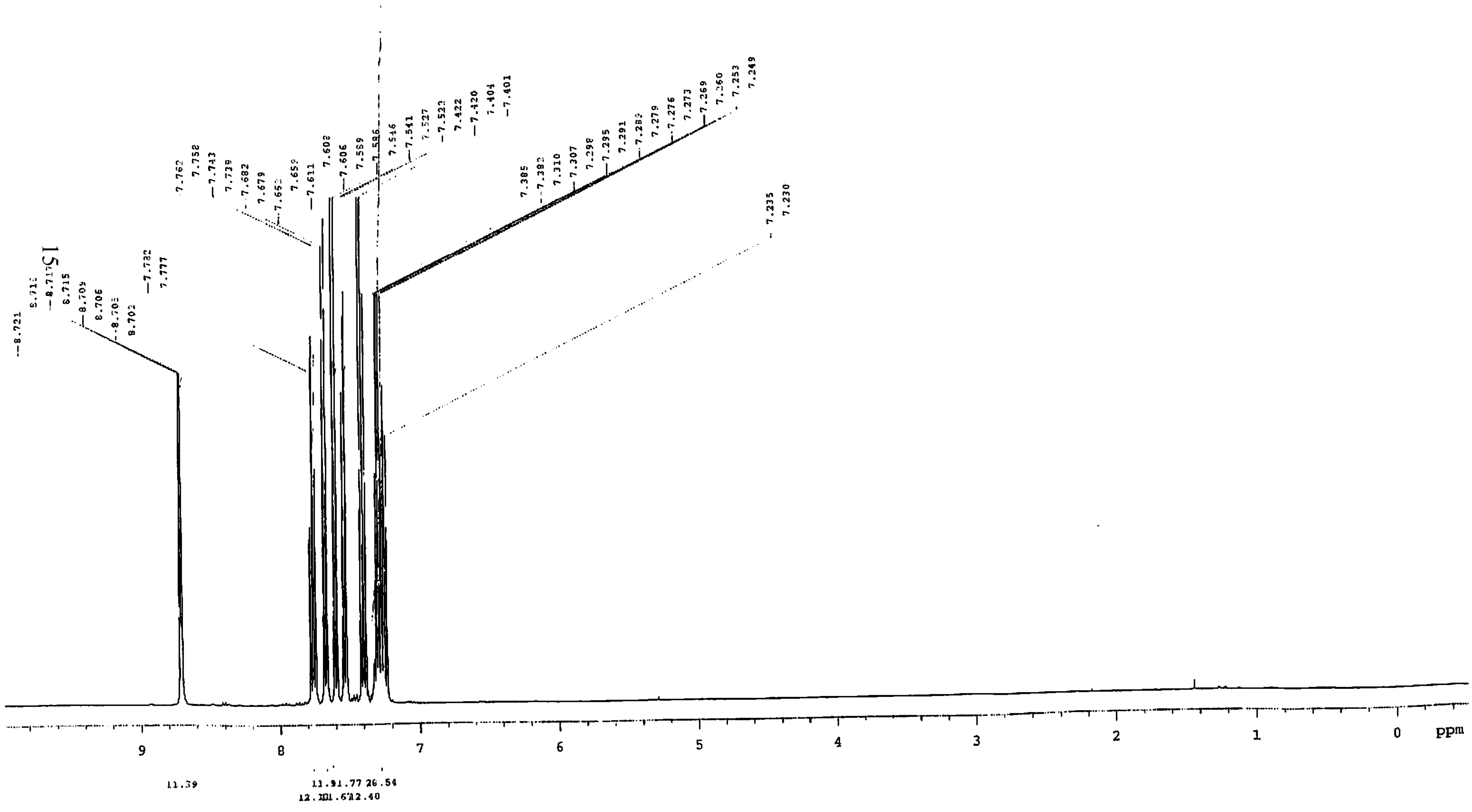
8.721
8.717
8.715
8.709
8.706
8.703
8.702
7.782
7.777
7.762
7.758
7.743
7.739
7.682
7.679
7.662
7.659
7.611
7.608
7.606
7.589
7.586
7.546
7.541
7.527
7.522
7.422
7.420
7.404
7.401
7.385
7.382
7.310
7.307
7.298
7.295
7.291
7.283
7.279
7.276
7.273
7.269
7.260
7.253
7.249
7.235
7.230
9
8
7
6
5
4
3
2
1
0
ppm
11.39
26.54
12.40

## $^{13}C$ NMR

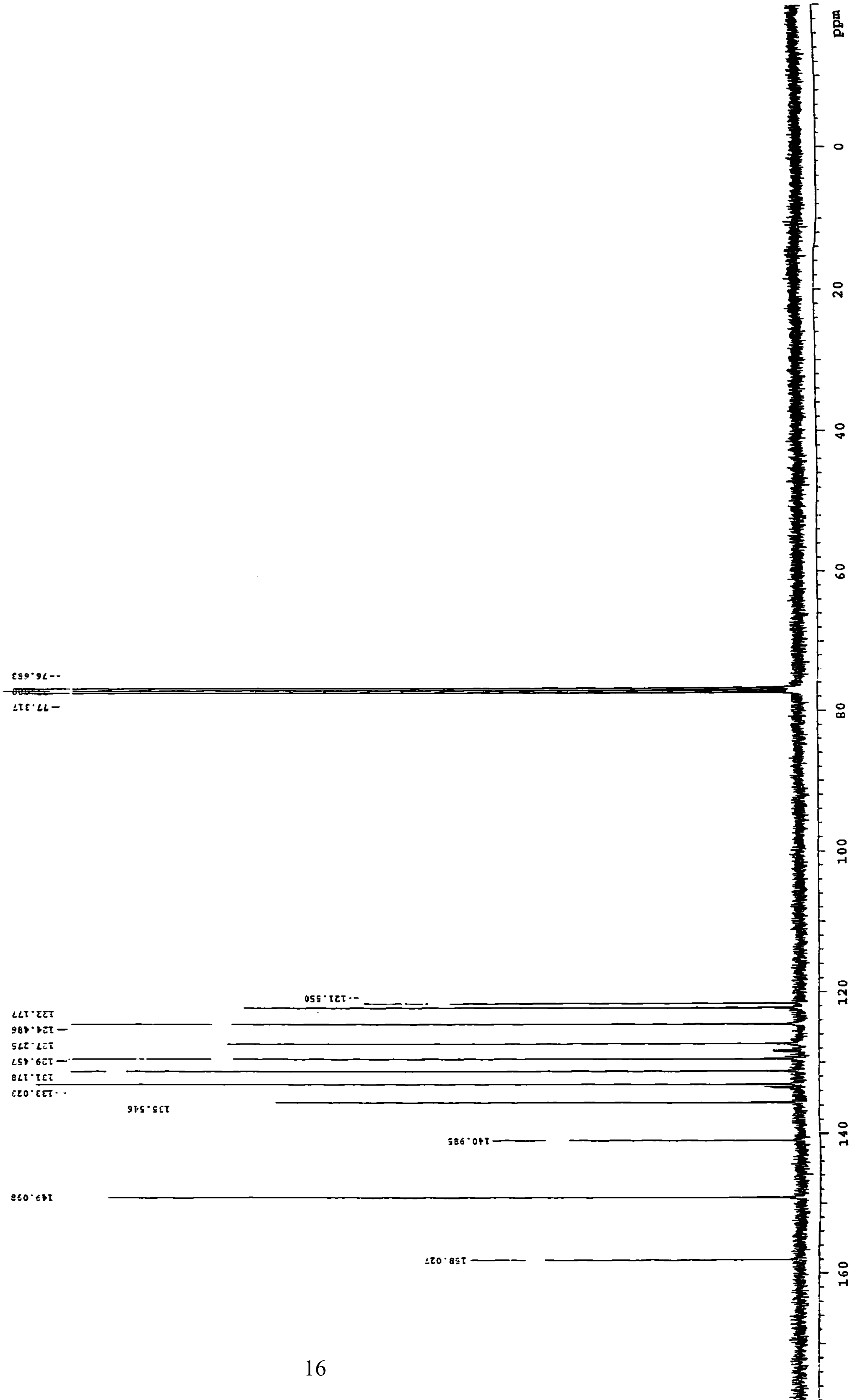

76.653
77.000
77.317
121.550
123.177
124.496
127.275
129.457
131.178
133.033
135.546
140.985
149.098
158.027
ppm
0
20
40
60
80
100
120
140
160


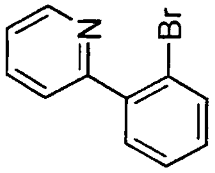

N
Br

$^1H$ NMR

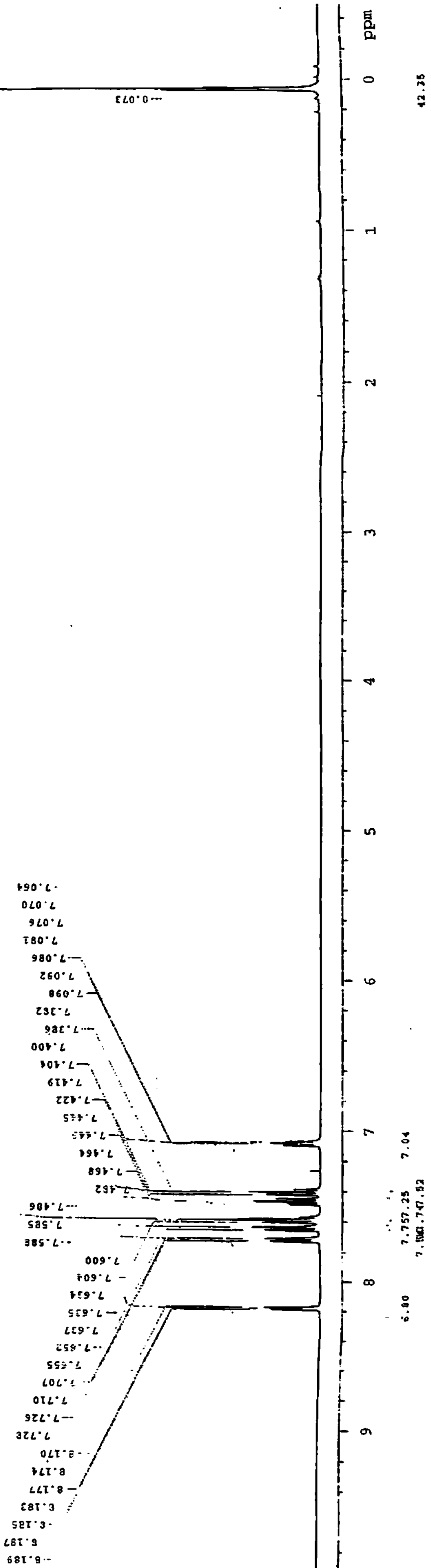

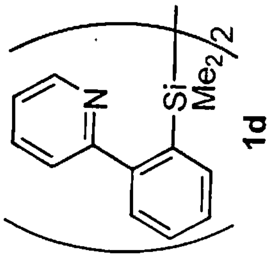

**1d**

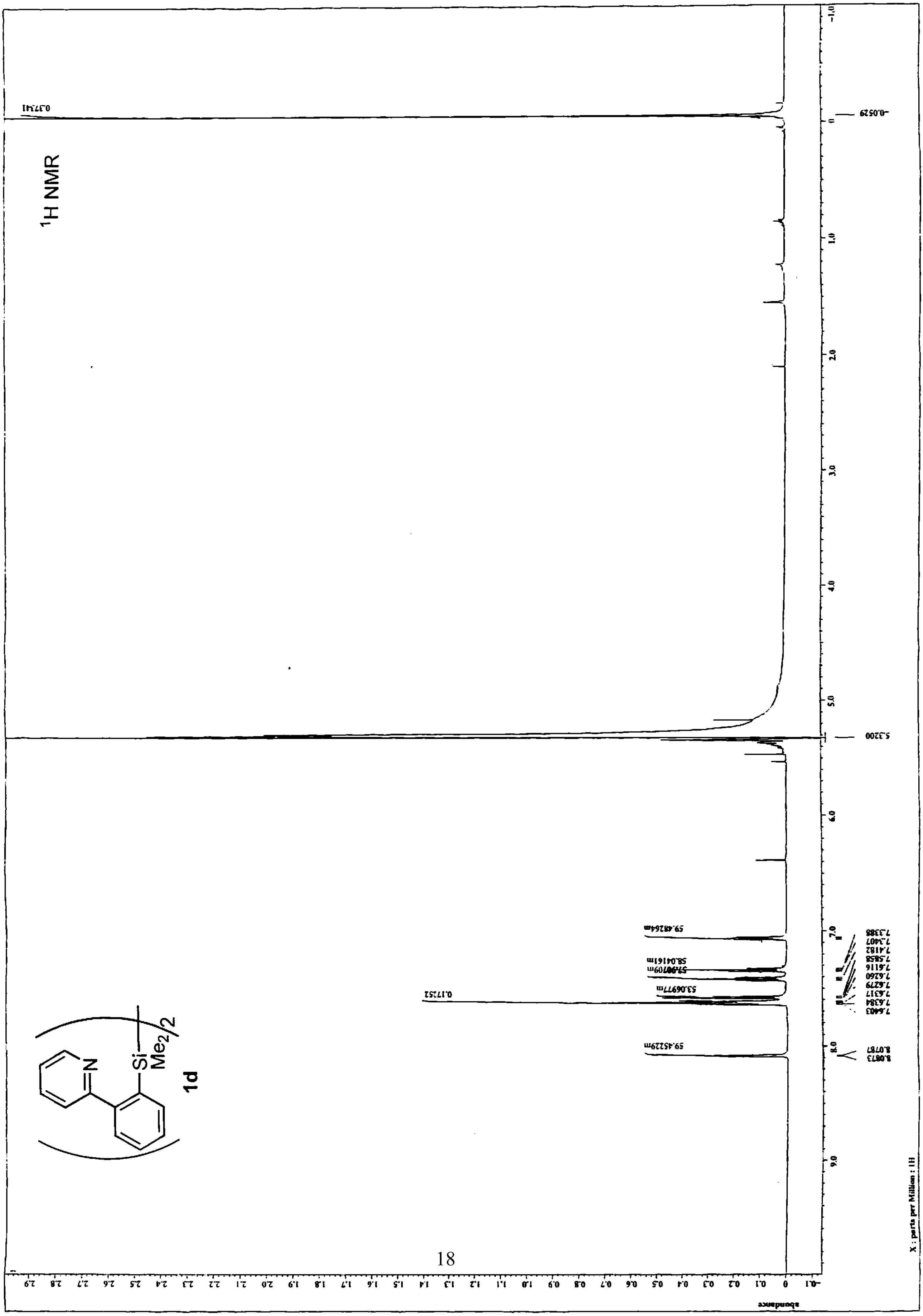

1H NMR
1d
Si
Me2
N
-0.0529
5.3200
7.3388
7.3407
7.4182
7.5858
7.6116
7.6260
7.6279
7.6317
7.6384
7.6403
8.0787
8.0873
X : parts per Million : 1H
abundance

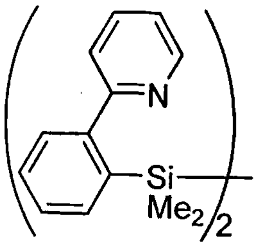

1d

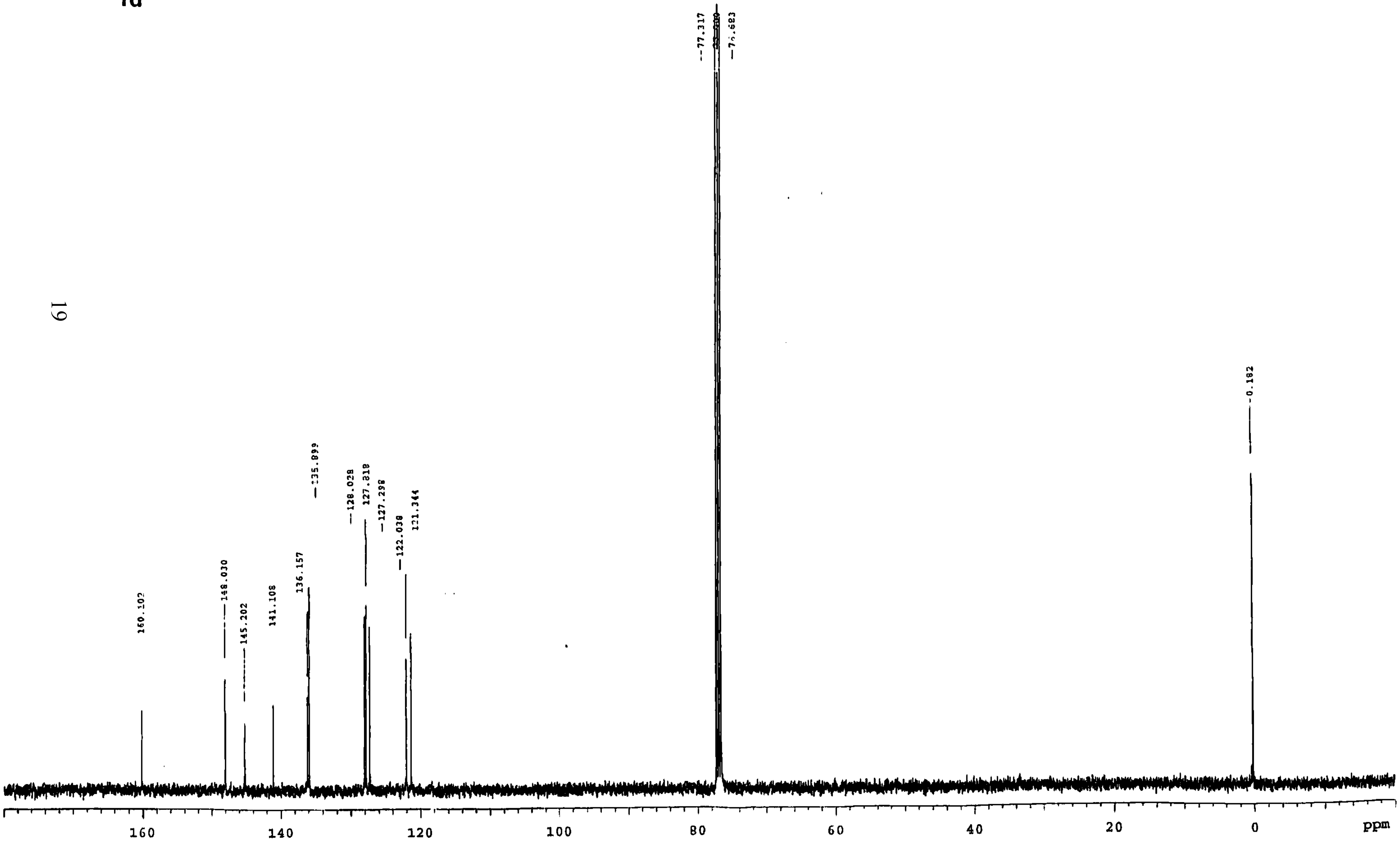

13C NMR
160.102
148.030
145.202
141.108
136.157
135.899
128.028
127.819
127.298
122.038
121.344
77.317
76.683
-0.182
160
140
120
100
80
60
40
20
0
ppm

$^{29}Si$ NMR

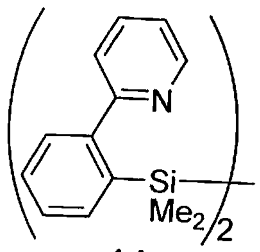

**1d**

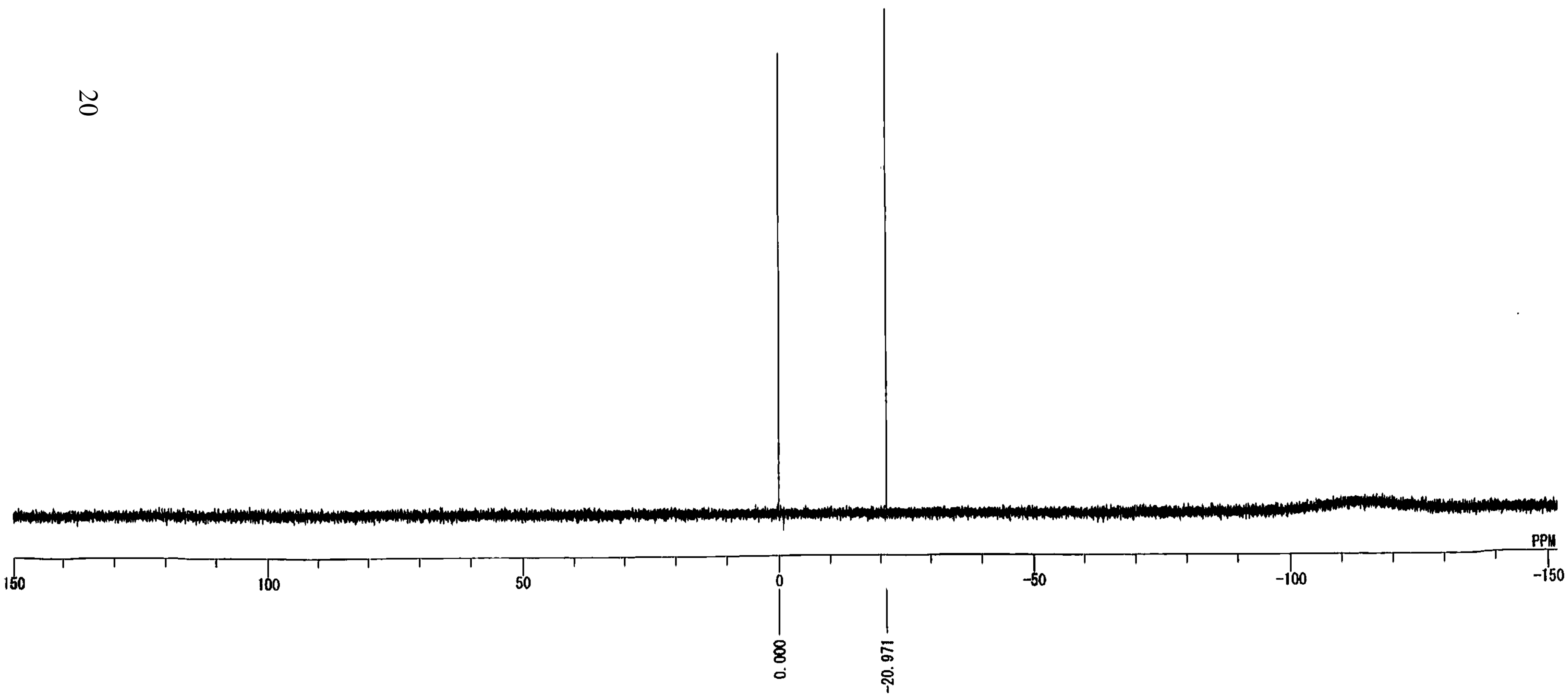

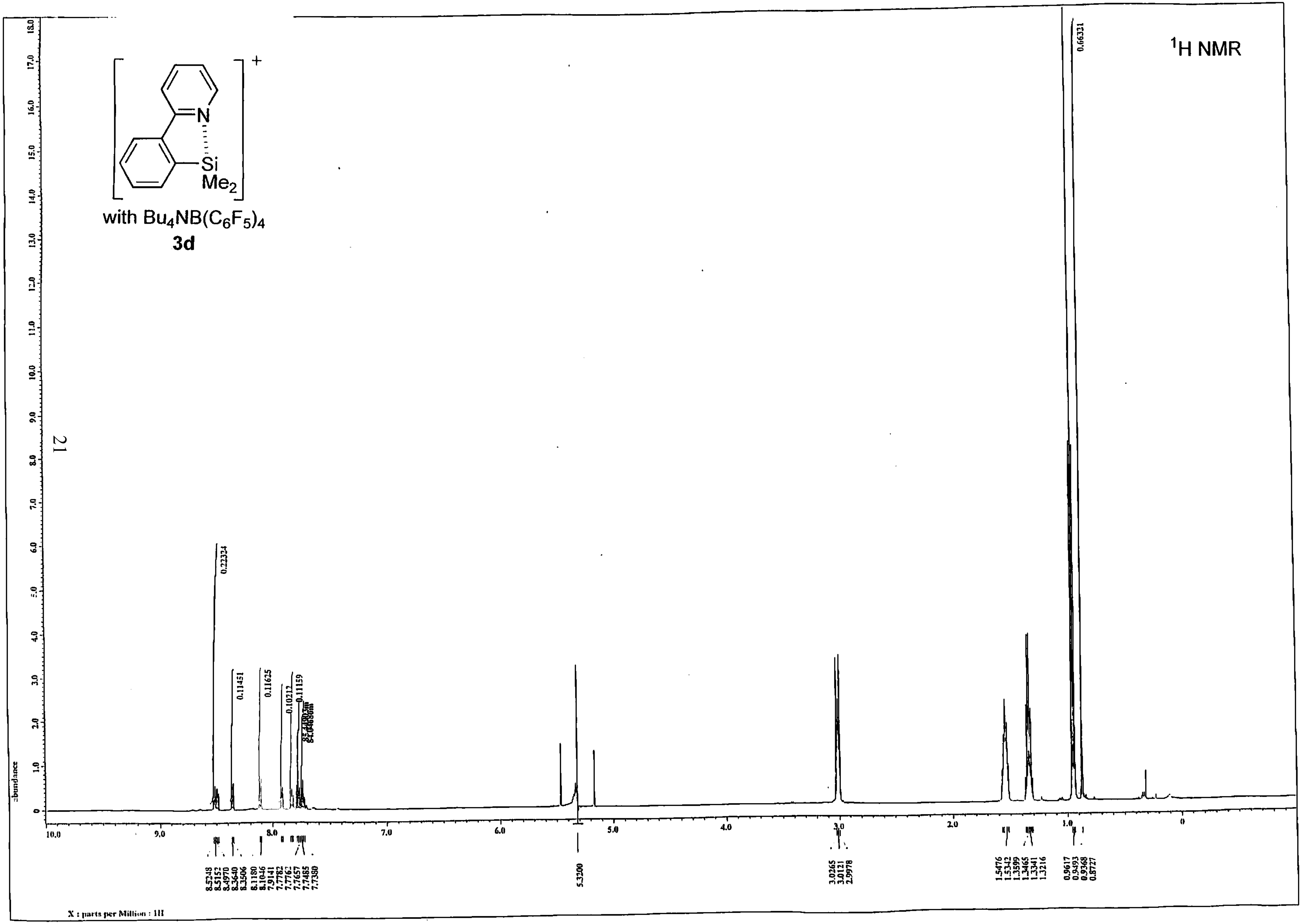

¹H NMR
with Bu₄NB(C₆F₅)₄
3d
0.66331
0.22324
0.11451
0.11625
0.10212
0.11159
8.5248
8.5152
8.4970
8.3640
8.3506
8.1180
8.1046
7.9141
7.7782
7.7762
7.7657
7.7485
7.7380
5.3200
3.0265
3.0121
2.9978
1.5476
1.5342
1.3599
1.3465
1.3341
1.3216
0.9617
0.9493
0.9368
0.8727
X : parts per Million : 1H
abundance

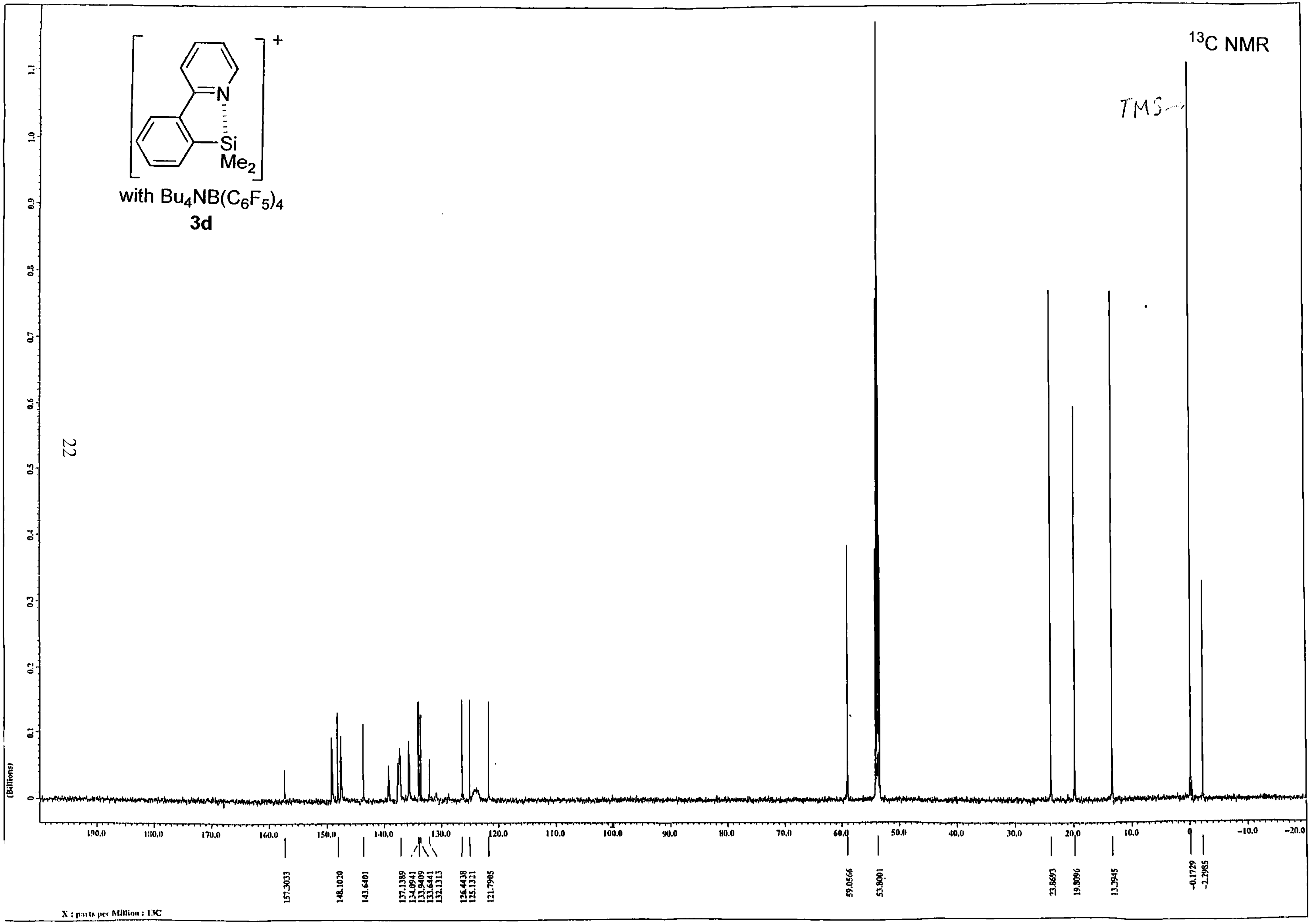

13C NMR
TMS
with Bu4NB(C6F5)4
3d
157.3033
148.1020
143.6401
137.1389
134.0941
133.9409
133.6441
132.1313
126.4438
125.1321
121.7905
59.0566
53.8001
23.8693
19.8096
13.3945
-0.1729
-2.2985
X : parts per Million : 13C
(Billions)

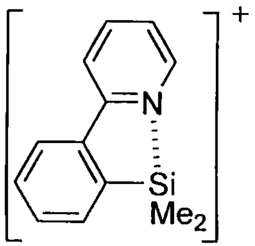

with $Bu_4NB(C_6F_5)_4$

**3d**

$^{29}Si$ NMR

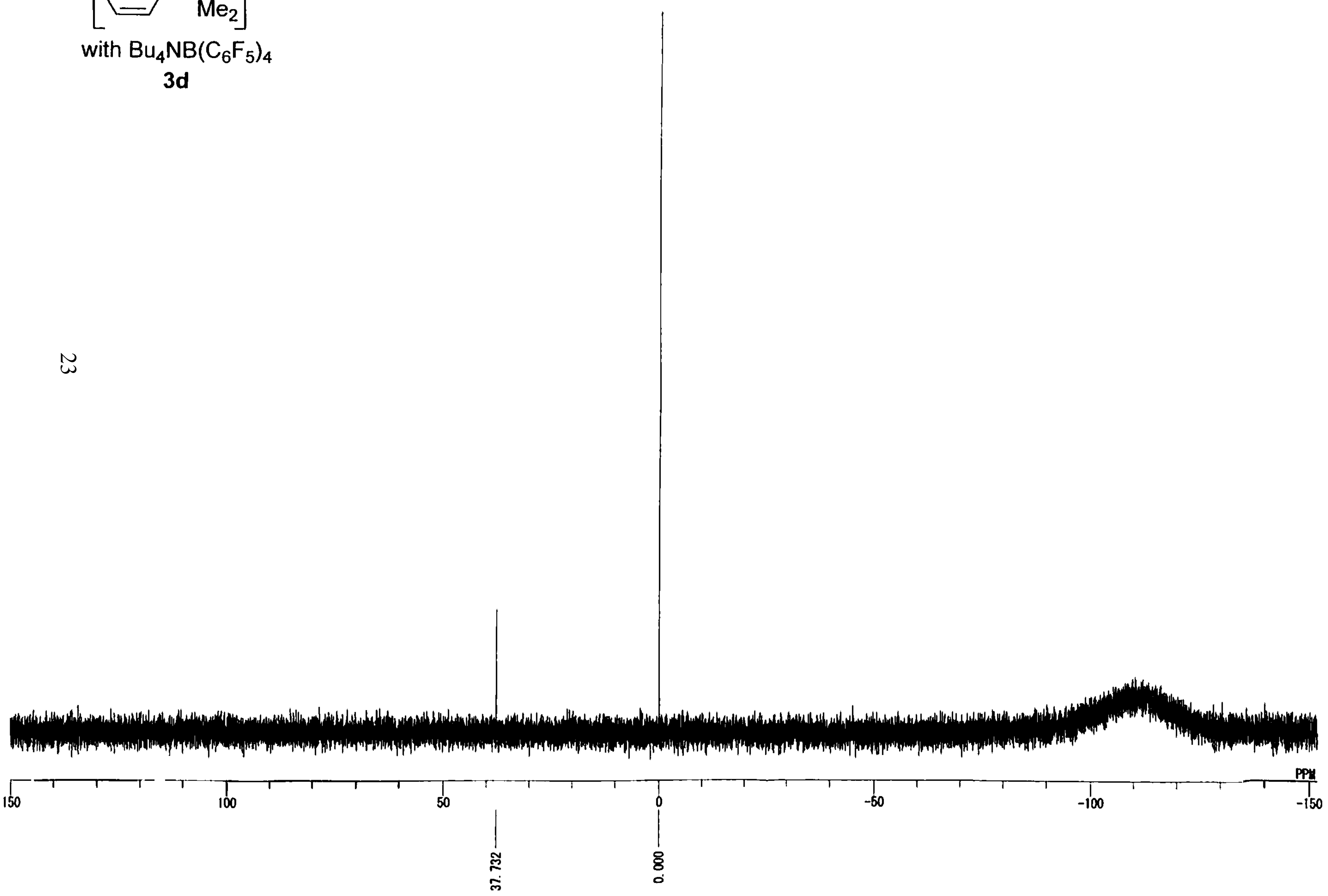

CSI-MS

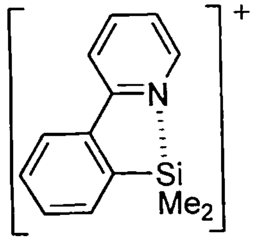

with $Bu_4NB(C_6F_5)_4$

**3d**

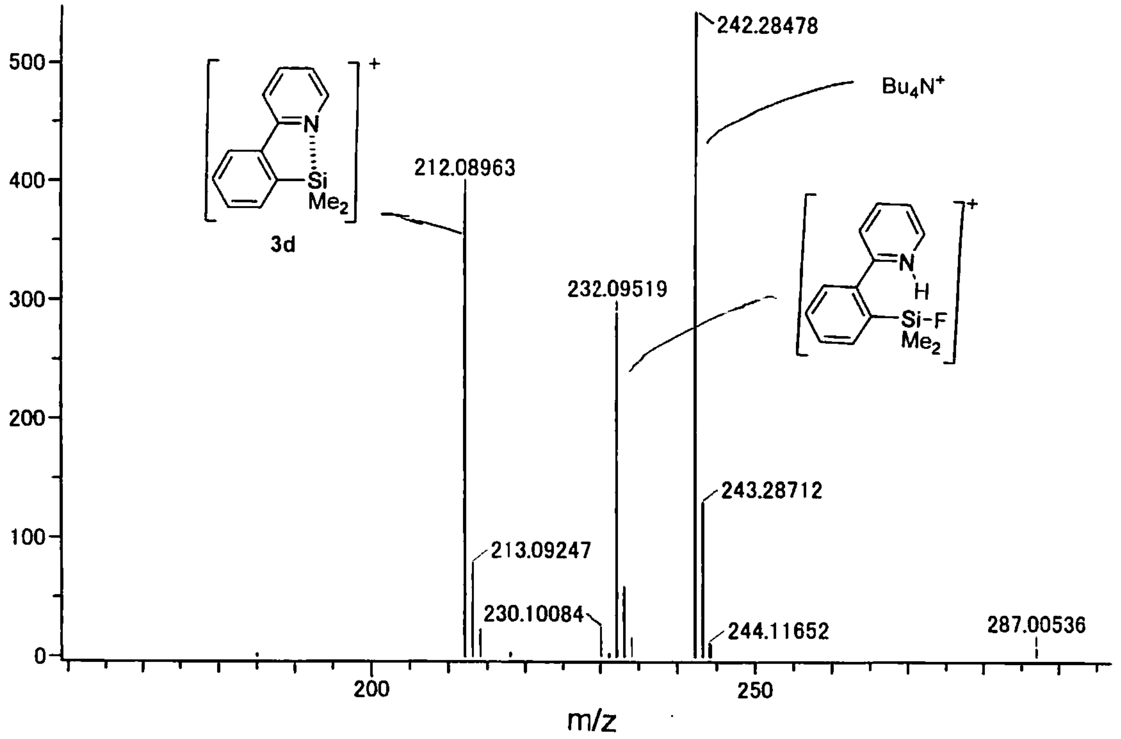

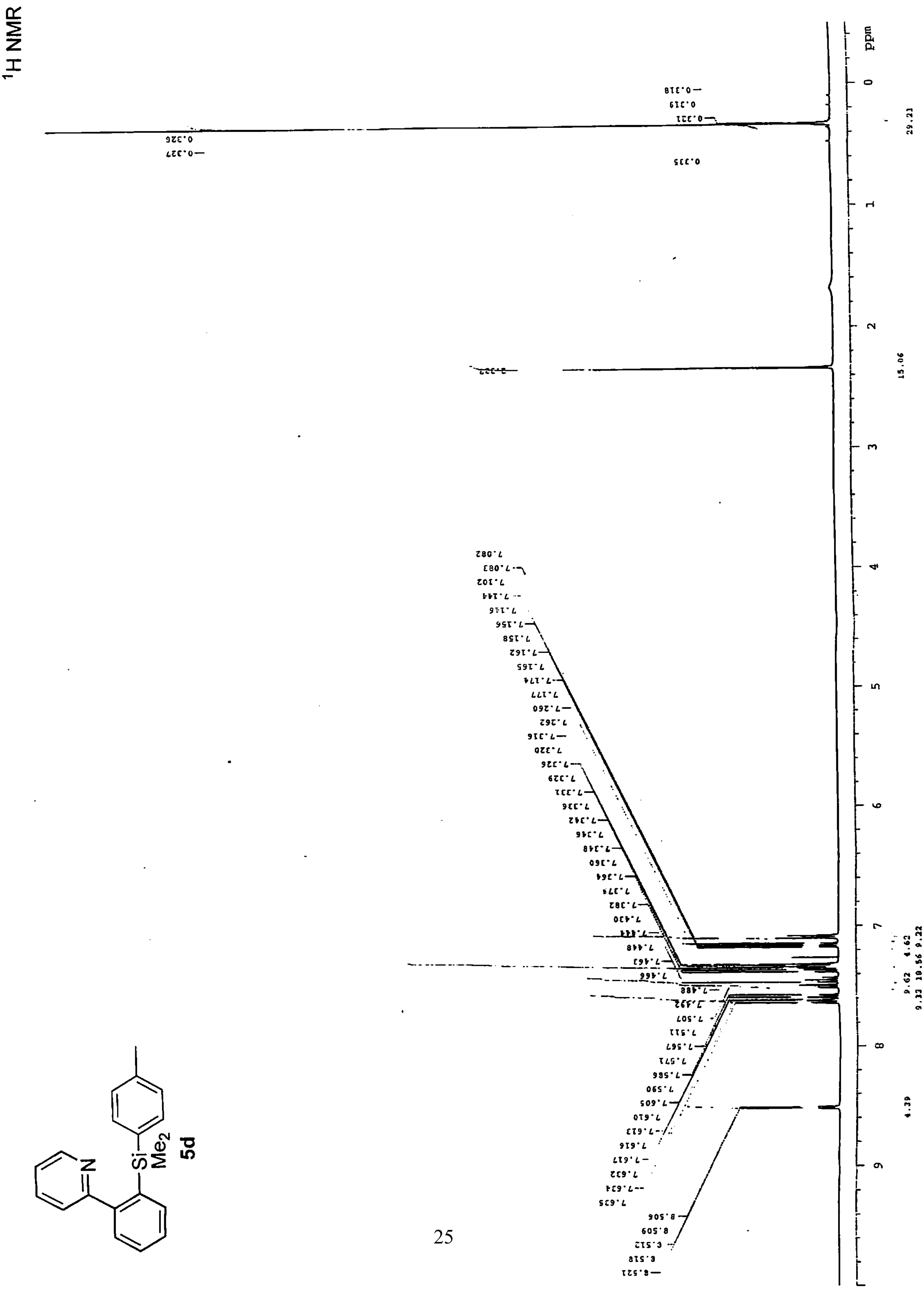

1H NMR
ppm
N
Si
Me2
5d

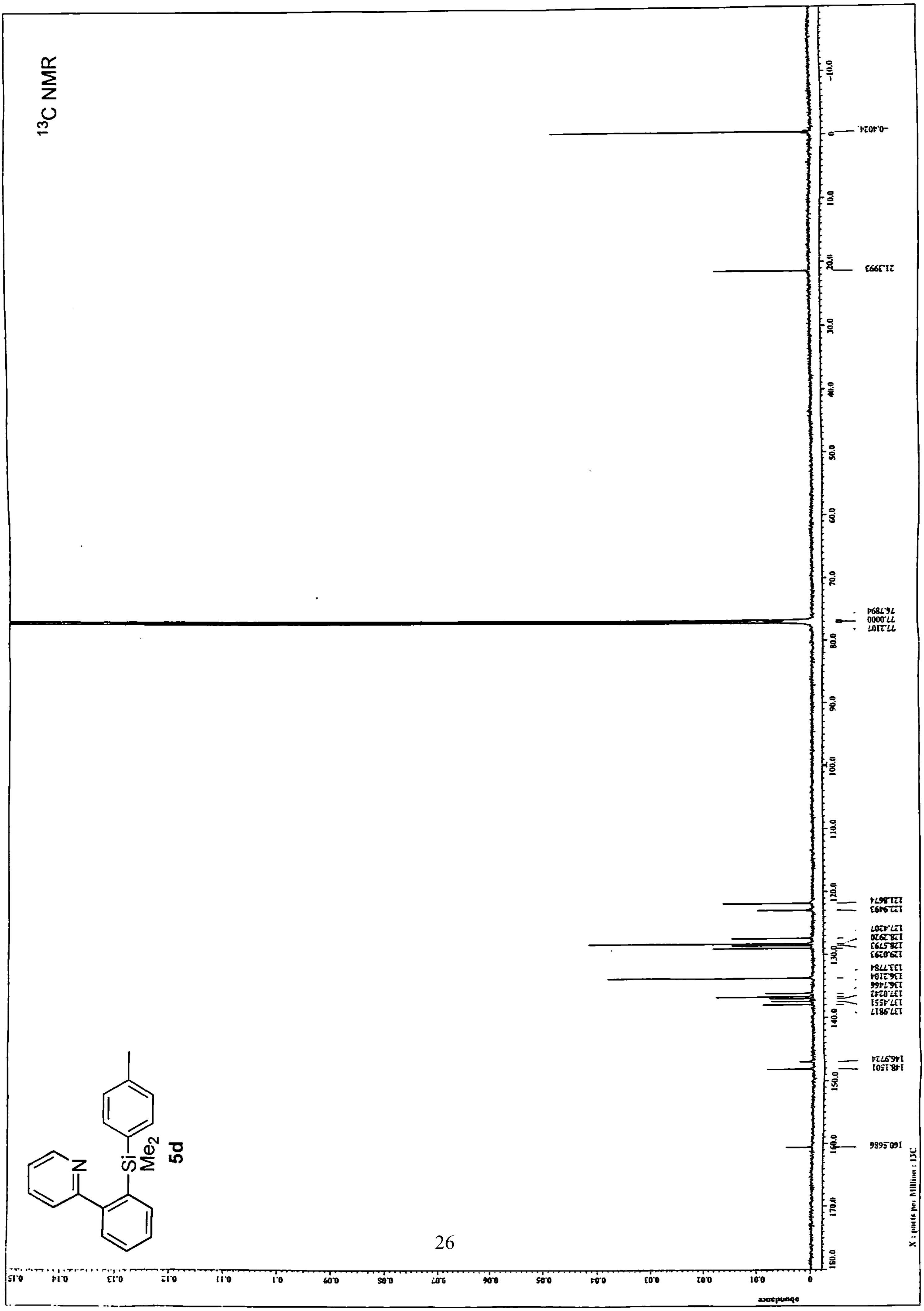

$^{13}C$ NMR
N
Si
$Me_2$
5d
X : parts per Million : 13C
abundance
